# Let-7 Represses Carcinogenesis and a Stem Cell Phenotype in the Intestine via Regulation of Hmga2

**DOI:** 10.1371/journal.pgen.1005408

**Published:** 2015-08-05

**Authors:** Blair B. Madison, Arjun N. Jeganathan, Rei Mizuno, Monte M. Winslow, Antoni Castells, Miriam Cuatrecasas, Anil K. Rustgi

**Affiliations:** 1 Division of Gastroenterology, Washington University School of Medicine, Saint Louis, Missouri, United States of America; 2 Department of Medicine, Washington University School of Medicine, Saint Louis, Missouri, United States of America; 3 Division of Gastroenterology, University of Pennsylvania Perelman School of Medicine, Philadelphia, Pennsylvania, United States of America; 4 Department of Medicine, University of Pennsylvania Perelman School of Medicine, Philadelphia, Pennsylvania, United States of America; 5 Department of Surgery, University of Pennsylvania Perelman School of Medicine, Philadelphia, Pennsylvania, United States of America; 6 Department of Genetics, Stanford University School of Medicine, Stanford, California, United States of America; 7 Gastroenterology Department, Hospital Clínic, CIBERehd, IDIBAPS, Barcelona, Catalonia, Spain; 8 Department of Pathology, Pharmacology and Microbiology, Hospital Clínic, CDB, University of Barcelona, Barcelona, Catalonia, Spain; 9 Department of Genetics, University of Pennsylvania Perelman School of Medicine, Philadelphia, Pennsylvania, United States of America; 10 Abramson Cancer Center, University of Pennsylvania Perelman School of Medicine, Philadelphia, Pennsylvania, United States of America; University of Maryland Medical School, UNITED STATES

## Abstract

Let-7 miRNAs comprise one of the largest and most highly expressed family of miRNAs among vertebrates, and is critical for promoting differentiation, regulating metabolism, inhibiting cellular proliferation, and repressing carcinogenesis in a variety of tissues. The large size of the Let-7 family of miRNAs has complicated the development of mutant animal models. Here we describe the comprehensive repression of all Let-7 miRNAs in the intestinal epithelium via low-level tissue-specific expression of the *Lin28b* RNA-binding protein and a conditional knockout of the *MirLet7c-2/Mirlet7b* locus. This ablation of Let-7 triggers the development of intestinal adenocarcinomas concomitant with reduced survival. Analysis of both mouse and human intestinal cancer specimens reveals that stem cell markers were significantly associated with loss of Let-7 miRNA expression, and that a number of Let-7 targets were elevated, including Hmga1 and Hmga2. Functional studies in 3-D enteroids revealed that Hmga2 is necessary and sufficient to mediate many characteristics of Let-7 depletion, namely accelerating cell cycle progression and enhancing a stem cell phenotype. In addition, inactivation of a single Hmga2 allele in the mouse intestine epithelium significantly represses tumorigenesis driven by Lin28b. In aggregate, we conclude that Let-7 depletion drives a stem cell phenotype and the development of intestinal cancer, primarily via Hmga2.

## Author Summary

Cancer develops following multiple genetic mutations (i.e. in tumor suppressors and oncogenes), and mutations that cooperate or synergize are often advantageous to cancer cell growth. To study how multiple genes might cooperate, it is usually informative to generate candidate mutations in cells or in mice. Large gene families, such as the Let-7 family, are difficult to silence or mutate because of the large amount of redundancy that exists between similar copies of the same gene; the mutation of one will often be masked or compensated by the continued function of others. In the mouse intestine we have achieved comprehensive depletion of all Let-7 miRNAs in this large multi-genic family through use of an inhibitory protein, called LIN28B, that specifically represses Let-7, and genetic inactivation of another gene cluster called *MirLet7c-2/Mirlet7b*. Mice with this comprehensive depletion of Let-7 develop intestinal cancers that resemble human colon cancers. Our further analysis identified another gene, *HMGA2*, downstream of this pathway that is critical to this outcome.

## Introduction

Micro-RNAs (miRNAs) are critical for tumor suppression, which is most notably revealed following genetic manipulation of *Dicer1*, an enzyme needed for miRNA processing, in which haplo-insufficiency of *Dicer1* and global reduction of miRNA levels significantly accelerates tumorigenesis [1,2]. Let-7 miRNAs comprise one of the largest and most highly expressed families of miRNAs, possessing potent anti-carcinogenic properties in a variety of tissues [3]. This activity is likely mediated via Let-7 repression of a multitude of onco-fetal mRNAs and other pro-proliferative and/or pro-metastatic targets, such as HMGA2, IGF2BP1, IGF2BP2, and NR6A1 [4–6]. Let-7 biogenesis is tightly regulated, revealed by the discovery of several proteins that regulate processing by DGCR8/DROSHA in the nucleus, and by DICER1 cleavage in the cytoplasm. Most notable are LIN28A and LIN28B, which are RNA-binding proteins that directly bind to and block the processing of Let-7 mRNAs [7,8]. LIN28A works in concert with TRIM25 and TUT4 to mediate terminal uridylation and subsequent degradation of immature precursor-Let-7 (pre-Let-7) miRNA molecules [9–11]. LIN28B appears to act by sequestering primary-Let-7 (pri-Let-7) miRNAs within the nucleolus to prohibit processing by DGCR8 and DROSHA [9]. The critical nature of maintaining sufficient levels of mature Let-7 miRNAs is reflected in the large number of studies that have found LIN28A or LIN28B up-regulated in human cancers, with expression often associated with an aggressive disease phenotype and/or predictive of poor outcomes [12–15]. LIN28B appears somewhat more frequently up-regulated than LIN28A in cancer, and may reflect the greater expression potential of LIN28B in adult tissues: LIN28B exhibits a later temporal pattern of expression in adult tissues such as the intestine, plays a greater role in post-natal growth, and can be re-activated by inflammation and NF-kappa-B [16–19].

In efforts of The Cancer Genome Atlas (TCGA) research consortia to define miRNA-mRNA associations across multiple different cancers (i.e. the pan-cancer initiative), the LIN28B:Let-7b interaction was identified as one of the most significant relationships discovered across nine different human malignancies [20]. The tight functional interplay between LIN28 proteins and Let-7 is delineated clearly, on biochemical and biological levels. However, Let-7 action appears dependent on the particular mRNA targets affected, although Let-7 represses de-differentiation in multiple contexts. For example, Let-7 regulates insulin-PI3K-mTOR signaling in muscle by inhibiting expression of *INSR*, *IGF1R*, and *IRS2* [21], yet can also inhibit mTORC1 without affecting insulin-PI3K signaling [22], whereas we have observed no effects on insulin-PI3K-mTOR signaling following depletion of Let-7 miRNAs in the small intestine [18]. Micro-RNAs have many targets, including both coding and non-coding mRNAs, and to address the functional impact of these miRNAs, one must dissect the cascade of integrated signals that ensue following alterations of a miRNA. Many studies have focused on *RAS* and *MYC* as cancer-relevant Let-7 targets, although recent high-throughput sequencing (mRNA-seq, miRNA-seq, and CLIP-seq) and meta-analyses indicate that these mRNA targets are not frequently regulated by Let-7, especially in the context of cancer [5,6,20,23]. Onco-fetal Let-7 targets such as *HMGA2* and *IGF2BP1-3* appear to be more frequently up-regulated in multiple contexts, across multiple tissues, and in association with somatic stem cell potential [4,5,20,24–29].

We have demonstrated that *LIN28B* is a potent driver of colorectal cancer (CRC) progression, cellular invasion, and in mouse models, a regulator of intestinal growth and tumorigenesis [15,18,30]. The exploration of Let-7-dependence through genetic manipulation in mouse models is currently untenable due to the large number of miRNA clusters, with 12 Let-7 genes located at 8 separate clusters on 7 different chromosomes. To circumvent this obstacle and elucidate the mechanistic roles of Let-7 miRNAs in intestinal tumorigenesis in a genetic mouse model we have combined a *Vil-Lin28b* ^*Low*^ (*Lin28b* ^*Lo*^) transgene with intestinal deletion of the *MirLet7c-2/Mirlet7b* bi-cistronic cluster (*Let-7* ^*IEC-KO*^) to achieve robust repression of all Let-7 miRNAs expressed in the intestinal epithelium. Concurrent deletion of the *MirLet7c-2/Mirlet7b* bi-cistronic cluster is necessary as Lin28b is unable to effectively target and inhibit processing of these specific Let-7 miRNAs [18].

These *Lin28b* ^*Lo*^ */Let-7* ^*IEC-KO*^ mice develop intestinal polyps with 100% penetrance and develop adenocarcinomas in the majority of animals, coincident with reduced survival. Examination of Let-7 targets in these tumors and in tumoroid cultures suggest that HMGA2 is likely playing a major role in driving carcinogenesis following Let-7 depletion, a novel *in vivo* finding. Furthermore, we find that tumorigenesis and a stem cell signature are driven by Let-7 depletion in mouse and human intestinal tumors, in which HMGA2 appears to play a functional role in reinforcing.

## Results

### Comprehensive Depletion of Let-7 miRNAs Leads to the Development of Intestinal Adenocarcinomas in Mice

**Fig 1:**
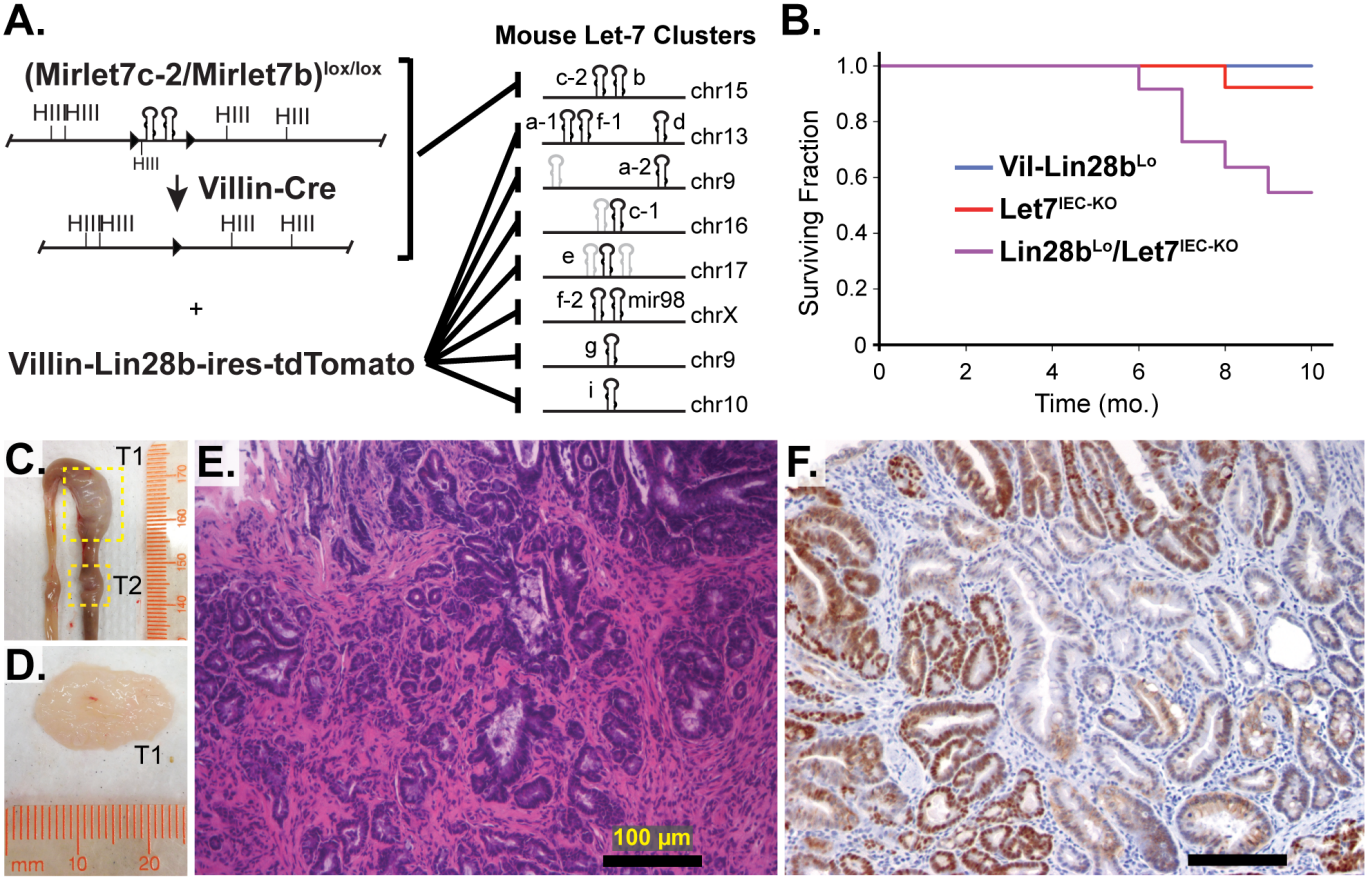
Comprehensive depletion of all Let-7 miRNAs leads to the development of intestinal adenocarcinomas. A) Schematic of the intestine-specific deletion of the *Mirlet7c-2/Mirlet7b* floxed locus via *Villin-Cre* and expression of Lin28b with a *Villin-Lin28b-ires-tdTomato* transgene, which repress all 8 of the Let-7 clusters. Let-7 miRNA genes are shown as black hairpins while non-let-7 miRNA genes are depicted as gray hairpins. B) Kaplan-Meier plot showing survival over 10 months. C) Representative small intestine from a *Lin28b* ^*Lo*^ */Let7* ^*IEC-KO*^ mouse containing two tumors, T1 and T2 (box outline with yellow dotted lines). D) Large tumor from (C) dissected with luminal side facing outward. E) H&E stained paraffin section of adenocarcinoma from a *Lin28b* ^*Lo*^ */Let7* ^*IEC-KO*^ mouse. F) Representative section of adenocarcinoma from a *Lin28b* ^*Lo*^ */Let7* ^*IEC-KO*^ mouse immunostained for β-catenin, showing a nuclear pattern of localization. Scale bars in (E) and (F) = 100 μm.

*Vil-Lin28b* ^*Low*^ mice and *Let7* ^*IEC-KO*^ mice were generated and described previously [18]. To generate compound mutant animals we used a low-expressing transgenic line (*Lin28b* ^*Lo*^ *)*, in which we could not detect measureable changes in either protein or mRNA levels of Let-7-independent Lin28b targets [18]. These compound *Lin28b* ^*Lo*^/*Let7* ^*IEC-KO*^ mice, exhibit depletion of all Let-7 miRNAs specifically in intestinal epithelial cells (IEC) achieved through deletion of the *MirLet7c-2/MirLet7b* locus and repression of all other Let-7 miRNAs through inhibition by Lin28b [18] (and Fig 1A). *Lin28b* ^*Lo*^/*Let7* ^*IEC-KO*^ mice thrived initially, with normal behavior and weight gain, but displayed significantly increased mortality and morbidity starting around 6 months of age, whereas neither *Vil-Lin28b* ^*Lo*^ nor *Let7* ^*IEC-KO*^ age-matched mice exhibited any overt phenotype (Fig 1B). Surviving *Lin28b* ^*Lo*^/*Let7* ^*IEC-KO*^ were sacrificed between 10 and 14 months of age and exhibited a significant incidence of adenomas and adenocarcinomas, restricted to the small intestine, with an average of 2.86 lesions per mouse and 100% penetrance (S1 Table and Fig 1C, 1D and 1E). Six of seven *Lin28b* ^*Lo*^/*Let7* ^*IEC-KO*^ mice developed invasive adenocarcinoma (S1 Table and Fig 1C, 1D and 1E). Tumors from mice also displayed nuclear localization of β-catenin (Fig 1F), indicative of constitutive activation of the Wnt signaling pathway. The severity of the *Lin28b* ^*Lo*^/*Let7* ^*IEC-KO*^ phenotype was substantially more dramatic than in *Vil-Lin28b* ^*Lo*^ or *Vil-Lin28b* ^*Med*^ mice (18). *Vil-Lin28b* ^*Med*^ mice express higher levels of Lin28b, have partially depleted Let-7 miRNAs and develop adenocarcinomas of the small intestine as do *Lin28b* ^*Lo*^ */Let7* ^*IEC-KO*^ mice but do not exhibit a phenotype as severe as *Lin28b* ^*Lo*^ */Let7* ^*IEC-KO*^ mice (18).

### Identification of Relevant Let-7 Target mRNAs in the Intestinal Epithelium and Tumors

**Fig 2:**
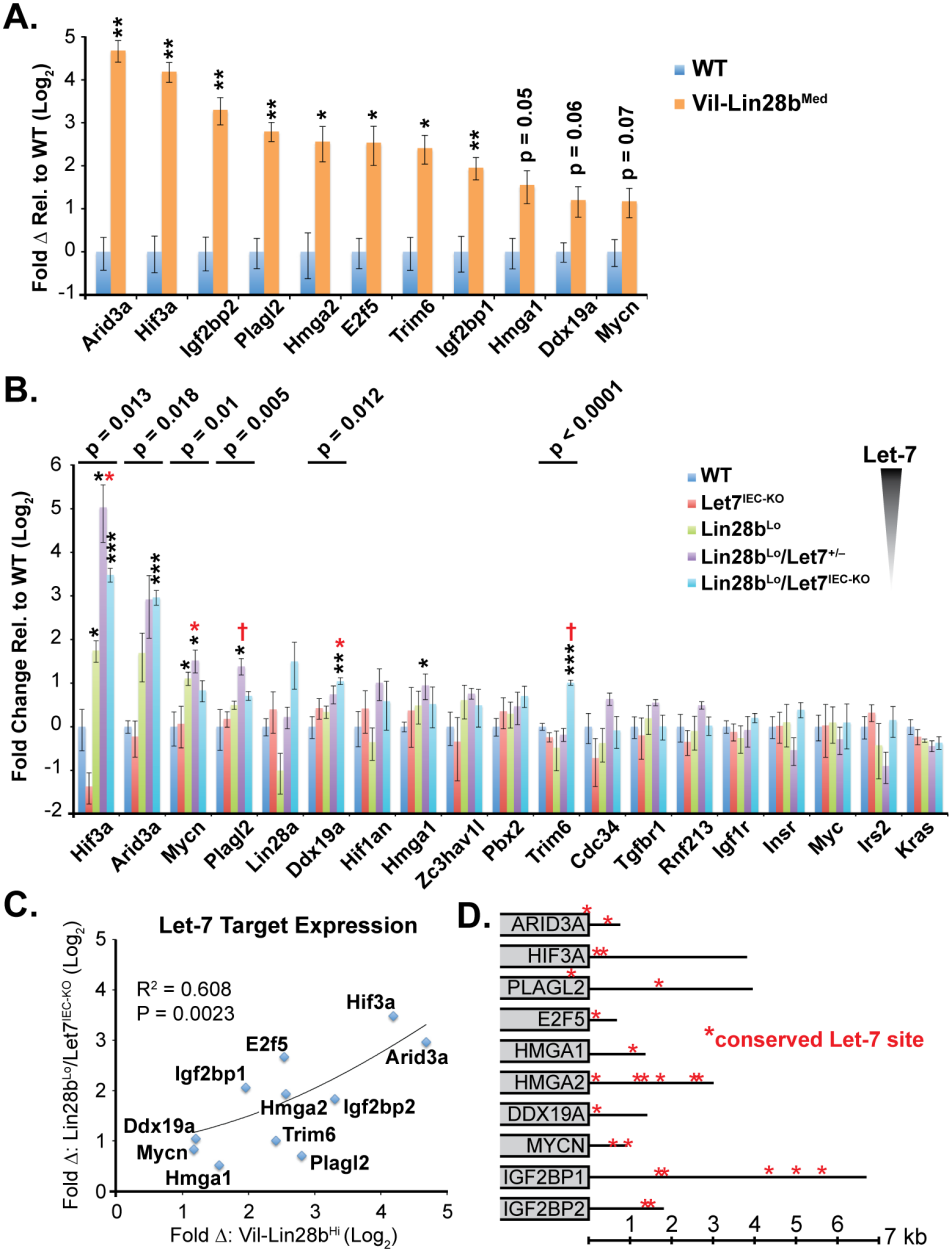
Quantification of Let-7 target mRNA levels in intestinal epithelium crypts. A) Expression of Let-7 target mRNA levels in small intestine crypts isolated from *wild-type* (WT) and *Vil-Lin28b* ^*Med*^ mice. B) Expression of Let-7 target mRNA levels in small intestine (jejunum) crypts isolated from *wild-type* (WT), *Vil-Lin28b* ^*Lo*^, *Let7* ^*IEC-KO*^, *Lin28b* ^*Lo*^ */Let7* ^*+/-*^, and *Lin28b* ^*Lo*^ */Let7* ^*IEC-KO*^ mice. C) Comparison of Let-7 target mRNA changes in small intestine crypts from *Vil-Lin28b* ^*Med*^ mice vs. *Lin28b* ^*Lo*^ */Let7* ^*IEC-KO*^ mice reveals similar expression changes in each model of Let-7 depletion, with significant correlation (Pearson correlation shown). Expression analysis was performed by Q-RT-PCR, normalized to *Hprt* and *Actb*, with n = 3 mice for each genotype at 12 weeks of age with error bars representing +/–the S.E.M. D) Identification of conserved Let-7 target genes in ten of eleven Let-7 target genes based upon TargetScan.org prediction. Student’s two-tailed T-tests were performed to determine significance with * p-value < 0.05, ** p-value < 0.01, and *** p-value < 0.001, relative to WT small intestine. One-way ANOVA standard weighted-means analysis was also performed in B, with p-values < 0.05 indicated above each gene. Tukey's HSD (honest significant difference) post-test was also performed in B, with samples p < 0.05 (red asterisk) and p < 0.01 (†) indicated, relative to mean of WT small intestine.

Let-7 targets were examined in small intestine crypts from *Vil-Lin28b* and *Lin28b* ^*Lo*^/*Let7* ^*IEC-KO*^ mice. RNA microarray expression analysis was previously performed on *Vil-Lin28b* ^*Med*^ total small intestine epithelia and we verified elevation of *Hmga1*, *Hmga2*, *Igf2bp1*, *Igf2bp2*, *E2f5*, *Acvr1c*, *Nr6a1*, *Hif3a*, *Arid3a*, *Plagl2*, *Trim6*, *Ddx19a*, and *Mycn* (Fig 2A and [18]). We also observed significant elevation of mRNAs for these Let-7 targets in crypts from small intestine epithelia from *Lin28b* ^*Lo*^/*Let7* ^*IEC-KO*^ (Fig 2B). Expression of all Let-7 targets also correlated significantly between *Lin28b* ^*Lo*^/*Let7* ^*IEC-KO*^ and *Vil-Lin28b* ^*Med*^ intestine crypts, with *Hmga2*, *Igf2bp2*, *Hif3a*, *Arid3a*, and *E2f5* being the most highly induced targets in both models (Fig 2C). All targets contained conserved Let-7 sites in the 3’UTR or coding sequence, except for *Trim6*, for which only the mouse mRNA possesses Let-7 sites (Fig 2D). In addition to our findings for HMGA2, IGF2BP1, and IGF2BP2, there is experimental evidence that HMGA1, E2F5, and ARID3A are also direct targets of Let-7 [6,31,32].

**Fig 3:**
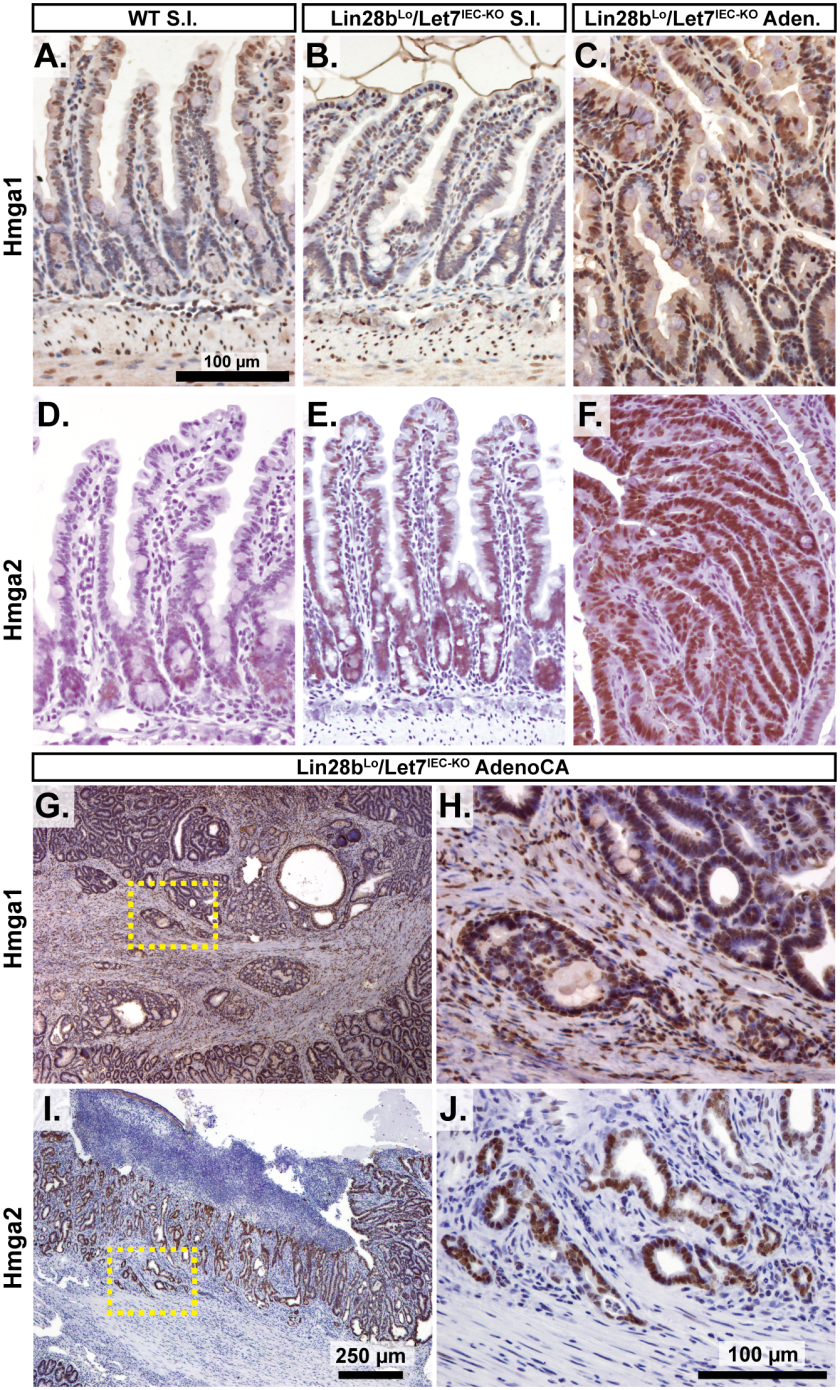
Hmga1 and Hmga2 proteins are increased in invasive areas of adenocarcinomas. Immunohistochemical staining for Hmga2 (A-C, G, H) and Hmga2 (D-F, I, J), in sections from WT small intestine (S.I.) (A, D), *Lin28b* ^*Lo*^ */Let7* ^*IEC-KO*^ S.I. (B, E), *Lin28b* ^*Lo*^ */Let7* ^*IEC-KO*^ adenoma (C, F), and *Lin28b* ^*Lo*^ */Let7* ^*IEC-KO*^ adenocarcinoma (AdenoCA) (G-J). An enlargement of a region containing invasive HMGA1-positive tumor cells from G (dotted yellow box) is pictured in H, while a region containing invasive HMGA2-positive tumor cells from I is likewise displayed in J. Pictures in A-F, H, and J are at same magnification (200x), with scale bar = 100 μm, while pictures in G and I are both at 40x, with scale bar = 250 μm.

To gain insight into the association of several Let-7 targets with tumorigenesis in vivo, we examined Hmga1, Hmga2, Arid3a, and Hif3a protein expression by immunostaining adenomas and adenocarcinomas, as well as adjacent normal tissue, from *Lin28b* ^*Lo*^ */Let7* ^*IEC-KO*^ mice. These targets exhibited little or modest up-regulation in normal small intestine epithelia of *Lin28b* ^*Lo*^ */Let7* ^*IEC-KO*^ mice, but dramatic increases in tumors (Fig 3A–3J and S3A–S3H Fig). Pathological assessment of the staining pattern revealed that Hmga1 and Hmga2 staining was most intense in areas of invasive adenocarcinoma (Fig 3G, 3H and 3I).

**Fig 4:**
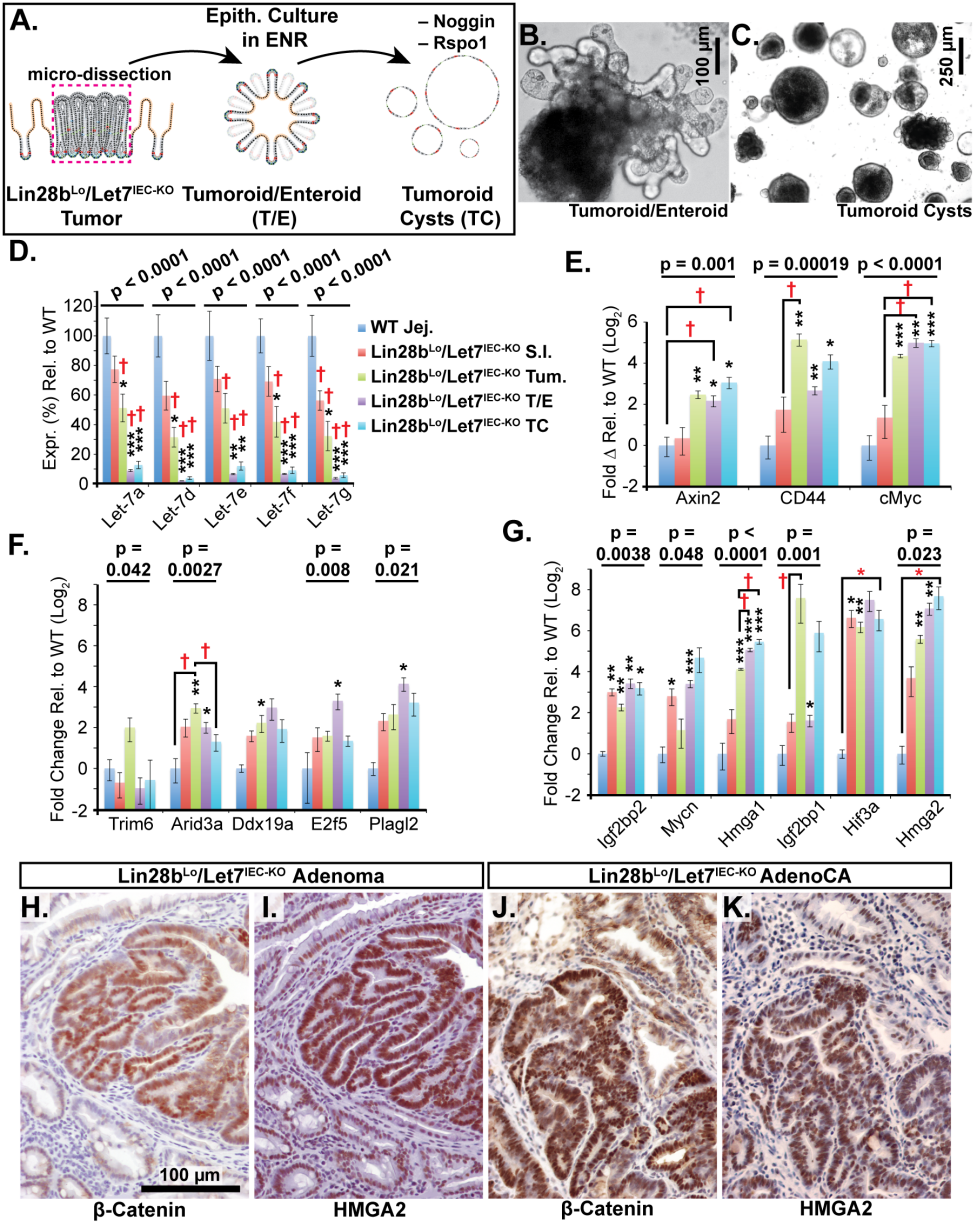
Identification of Let-7 targets up-regulated specifically in transformed cells from intestinal adenocarcinomas. A) Schematic of experimental procedure where tumors were micro-dissected from the small intestine (S.I.) of *Lin28b* ^*Lo*^ */Let7* ^*IEC-KO*^ mice and cultured as epithelial tumoroid/enteroids (T/E), grown in ENR medium, and tumoroid cysts (TC), grown in medium lacking Noggin and Rspo1. B) Typical tumoroid grown in ENR medium. C) Tumoroid cysts grown in basal medium containing EGF. D) Let-7 miRNAs are repressed consistently in tumoroid/enteroids (TE) and tumoroid cysts (TC). E) Wnt (Tcf4/β-catenin) target genes Axin2, CD44 and cMyc mRNAs were up-regulated in tumors, T/E, and TC. F) Transcripts with highest expression in tumor or tumoroid, but tend to be down-regulated in tumoroid cysts. G) Transcripts that maintain high expression and/or are increased in tumoroid cysts. Note logarithmic scale where Hmga2 mRNA is induced approximately 200-fold in TC compared to wild-type S.I. Immunostaining in *Lin28b* ^*Lo*^ */Let7* ^*IEC-KO*^ adenomas (H, I) and adenocarcinomas (J, K) revealed that nuclear β-catenin (H, J) and Hmga2 (I, K) are often co-expressed at high levels. Expression analysis was performed by Q-RT-PCR, normalized to *Hprt* and *Actb*, with n = 3–4 for each tissue/organoid with error bars representing +/–the S.E.M. Student’s two-tailed T-tests were performed to determine significance with * p-value < 0.05, ** p-value < 0.01, and *** p-value < 0.001, relative to wild-type small intestine. One-way ANOVA standard weighted-means analysis was also performed in D-G, with p-values < 0.05 indicated above each gene. Tukey's HSD (honest significant difference) post-test was also performed in D-G, with samples p < 0.05 (red asterisk) and p < 0.01 (†) indicated, relative to mean of WT small intestine (jej.).

We next examined Let-7 targets that might mediate programs of tumorigenesis in *Lin28b* ^*Lo*^/*Let7* ^*IEC-KO*^ mice in the context of tumors and cellular transformation. To model intestinal epithelial carcinogenesis we developed a 3-D culture model to examine only the epithelium and to select transformed tumor cells (Fig 4A). Enteroids derived from CRC tumors appear to faithfully recapitulate the major expression signatures of un-manipulated whole tumors [33]. To pursue this, we micro-dissected and dissociated adenocarcinomas from *Lin28b* ^*Lo*^/*Let7* ^*IEC-KO*^ mice and cultured “tumoroids” from these lesions in medium supplemented with EGF, Noggin, and Rspo1, as described previously for enteroid culture [34]. These tumoroid/enteroids (T/E) resembled normal small intestine enteroids (Fig 4B) and are likely a mixture of different cell types, but upon withdrawal of Noggin and Rspo1, a small population of growth-factor independent cells expanded into tumoroid cysts (TC) (Fig 4C), which likely possess cell-autonomous activation of Wnt signaling and Noggin-independent resistance to BMP signaling. Quantification by Taqman RT-PCR confirmed that Let-7 miRNAs are severely repressed in tumoroid/enteroids and transformed tumoroid cysts (Fig 4D). Tumors and tumoroids, but not normal tissue from *Lin28b* ^*Lo*^/*Let7* ^*IEC-KO*^ mice, also exhibited up-regulation of Wnt target genes *Axin2*, *CD44*, and *cMyc* (Fig 4E), suggesting spontaneous and constitutive activation of Wnt signaling. Analysis of Let-7 target mRNAs revealed two basic patterns of expression, with one group displaying expression highest in intact tumors or tumoroids/enteroids (Fig 4F). The other group displayed increasing or plateauing expression, with higher levels in tumoroid/enteroids or tumoroid cysts (Fig 4G). In this latter group we find known and suspected oncogenes, such as *Hmga1*, *Hmga2*, *Igf2bp1*, *Igf2bp2*, and *Mycn*. As Hmga2 appeared to exhibit pronounced up-regulation (>200-fold) in the tumoroid/enteroid and tumoroid cyst populations, and increased staining in invasive areas of adenocarcinomas (Fig 3H), we evaluated Hmga2 co-localization with nuclear β-catenin in mouse tumors, to assay potential coincident activation of canonical Wnt signaling with nuclear Hmga2. Immunostaining in both adenomas and adenocarcinomas from *Lin28b* ^*Lo*^/*Let7* ^*IEC-KO*^ mice revealed frequent and intense co-staining of Hmga2 with nuclear β-catenin (Fig 4H–4K). This pattern of co-staining was not observed for Hmga1, Arid3a, or Hif3a.

### Let-7 Down-Regulation and HMGA2 Up-Regulation Are Associated with a Stem Cell Signature in Intestinal Cancers in Humans and *Lin28b* ^*Lo*^/*Let7* ^*IEC-KO*^ Mice

**Fig 5:**
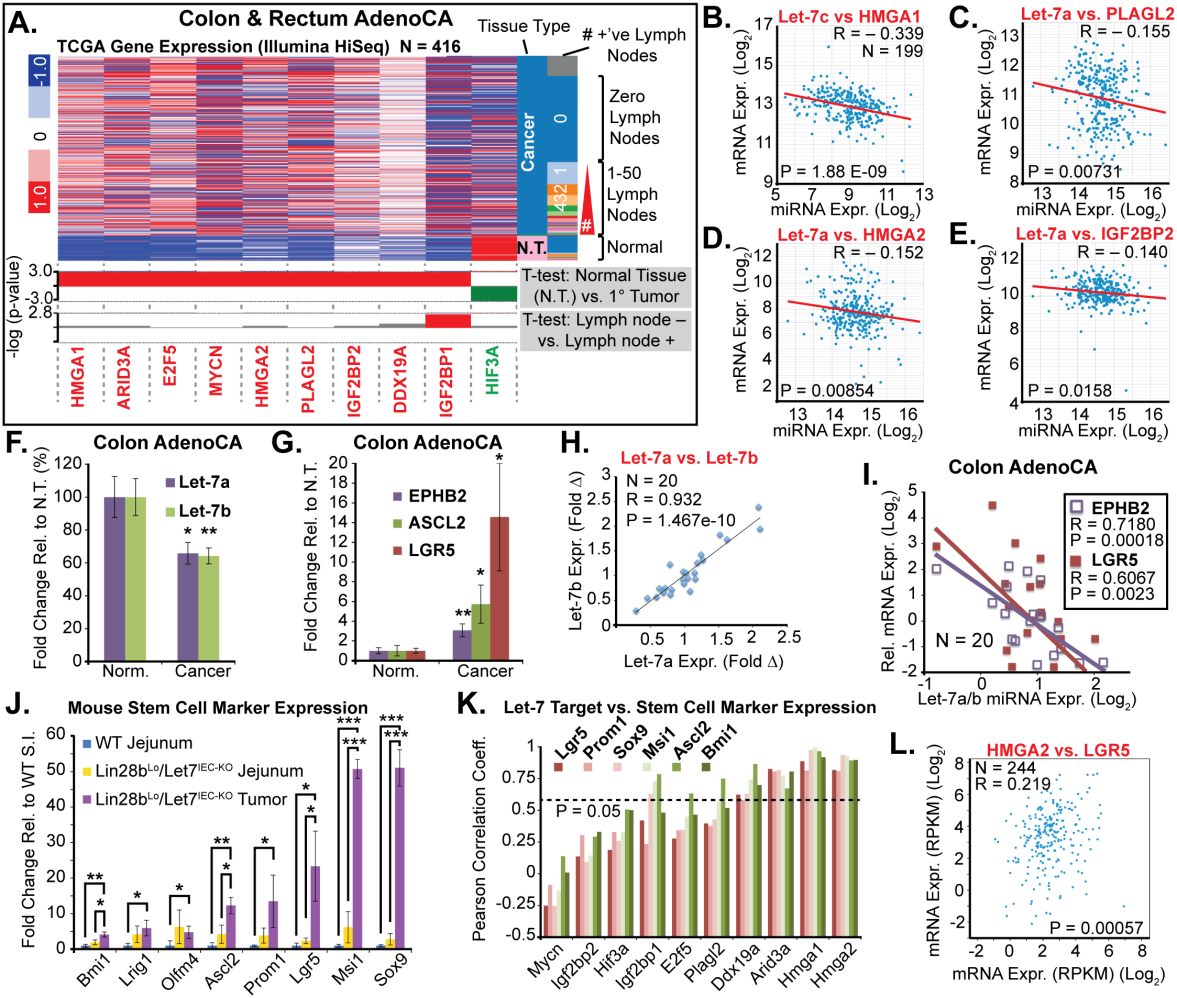
Let-7 and HMGA2 are associated with a stem cell signature in intestinal adenocarcinomas. A) Heat map of TCGA mRNA-seq colon and rectal adenocarcinoma dataset from UCSC Cancer Genome Browser (genome-cancer.ucsc.edu) comparing expression of Let-7 target mRNAs in normal tissue (N.T.) vs. cancer. Significant up-regulation (red) or down-regulation (green) is indicated below heatmap in plots of the-log(p-value) of Benjamini-Hochberg-corrected T-tests on the y-axis. T-test results are shown for expression in tumors vs. N.T. and in tumors associated with at least one lymph node metastases vs. tumors with no associated lymph node metastases. Inverse relationships for Let-7 and target mRNAs could be discerned by plotting miRNA-seq data against mRNA-seq data for Let-7c vs. *HMGA2* (B), Let-7a vs. *PLAGL2* (C), Let-7a vs. *HMGA2* (D), and Let-7a vs. *IGF2BP2* (E). F) Taqman QPCR for mature Let-7a and Let-7b miRNAs in a cohort of colon adenocarcinomas (N = 20) indicates that Let-7a and Let-7b are down-regulated. G) Intestinal epithelial stem cell markers *EPHB2*, *ASCL2*, and *LGR5* are significantly up-regulated in colon cancer vs. normal adjacent tissues. H) Mature Let-7a and Let-7b levels are tightly correlated in these tissue specimens. I) Let-7a and Let-7b levels are inversely proportional to mRNA levels of stem cell markers *EPHB2* and *LGR5*, suggesting that Let-7 may repress a stem cell signature. J) Expression of stem cell markers is dramatically up-regulated in *Lin28b* ^*Lo*^ */Let7* ^*IEC-KO*^ tumors, relative to WT jejunum, with a trend for up-regulation in *Lin28b* ^*Lo*^ */Let7* ^*IEC-KO*^ jejunum, relative to WT. K) Comparison of stem cell marker expression and Let-7 target mRNA expression levels in WT jejunum, *Lin28b* ^*Lo*^ */Let7* ^*IEC-KO*^ jejunum, and *Lin28b* ^*Lo*^ */Let7* ^*IEC-KO*^ tumors by linear regression yielded Pearson correlation coefficients, with *Arid3a*, *Hmga1*, and *Hmga2* correlating very highly with expression of stem cell markers. L) HMGA2 and LGR5 expression from the TCGA mRNA-seq colon and rectal adenocarcinoma dataset exhibit significant positive correlation. Expression analysis (F-K) was performed by QPCR, normalized to *Hprt* and *Actb*, with n = 3–4 for each mouse genotype with error bars representing +/–the S.E.M. Human QPCR was normalized to *PPIA* and *B2M*, with error bars representing +/–the S.E.M. Student’s two-tailed T-tests were performed to determine significance with * p-value < 0.05, ** p-value < 0.01, and *** p-value < 0.001.

To extrapolate relevance to human CRC from these mouse models, we examined expression data from human samples from The Cancer Genome Atlas (TCGA) [35] by querying for expression of Let-7 target mRNAs, with a focus on targets that exhibited significant up-regulation in either *Vil-Lin28b* ^*Med*^ or *Lin28b* ^*Lo*^/*Let7* ^*IEC-KO*^ mouse models (namely, *ARID3A*, *PLAGL2*, *HMGA1*, *HMGA2*, *MYCN*, *IGF2BP1*, *IGF2BP2*, and *E2F5*). We examined a cohort of 416 CRC patients from a TCGA dataset and found that all transcripts except *HIF3A* mRNA were significantly elevated in cancer tissue compared to expression levels in normal tissues (Fig 5A). *IGF2BP1* expression in primary tumors was also associated with an increased likelihood of having nodal metastases (Fig 5A). Levels of HMGA1, HMGA2, PLAGL2, IGF2BP2, E2F5, and ARID3A transcripts were also inversely proportional to levels of Let-7 miRNA by examination of a cohort of 199 CRC patients from the TCGA Pan-Cancer analysis project [20] (Fig 5B–5E and S1D–S1I Fig). Since Let-7a and Let-7b appear to be the most highly expressed Let-7 miRNAs in normal colonic epithelium, and are significantly depleted in CRC specimens [20,30] (S1A, S1B and S1C Fig), we examined these miRNAs in a subset of colon cancer specimens. We also compared their expression with the crypt-base-columnar (CBC) stem cell markers EPHB2, ASCL2, and LGR5, which are markers of stem cells in human intestine/colon and CRC, and are associated with aggressive CRC [36]. We found that Let-7a and Let-7b were significantly down-regulated in CRC specimens, while stem cell markers were significantly up-regulated (Fig 5F and 5G). Let-7a and Let-7b levels were also correlated tightly, suggesting co-regulation (Fig 5H), and were also inversely proportional to the expression of the stem cell markers EPHB2 and LGR5 (Fig 5I). This suggests provocatively that Let-7a and Let-7b depletion may contribute to a stem cell phenotype in the intestine, and perhaps CRC.

To further examine this relationship we evaluated small intestine stem cell markers in wild-type intestine, *Lin28b* ^*Lo*^/*Let7* ^*IEC-KO*^ intestine, and in tumors from *Lin28b* ^*Lo*^/*Let7* ^*IEC-KO*^ mice. In normal adjacent tissue we observed a trend toward increased expression of multiple stem cell markers in *Lin28b* ^*Lo*^/*Let7* ^*IEC-KO*^ small intestine (Fig 5J). In contrast, tumors from *Lin28b* ^*Lo*^/*Let7* ^*IEC-KO*^ mice exhibited a pronounced up-regulation of all stem cell markers assayed, including *Bmi1*, *Lrig1*, *Olfm4*, *Ascl2*, *Prom1*, *Lgr5*, *Msi1*, and *Sox9* (Fig 5J), perhaps suggesting an expansion of CBC and +4 stem cell-like compartments. While Let-7a and Let-7b depletion and increased expression of stem cell markers may appear to be a general feature of colon cancer, our discovery of a relationship between expression of Let-7 and stem cell markers suggests a functional connection. To examine a possible relationship between Let-7 target mRNAs and stem cell markers, we evaluated co-expression in mouse samples (from Fig 5I) and found that Hmga1 and Hmga2 had very high correlation with all of the markers we examined (Fig 5K). Likewise, in human CRC samples the expression of HMGA2 directly correlates with LGR5 levels (Fig 5L).

### HMGA1 or HMGA2 Expression Is Associated with Aggressive CRC in Patients

In order to explore any disease relevance connecting HMGA1 and HMGA2 expression and tumor phenotype, we stained CRC tumor tissue arrays for these proteins and evaluated expression in relationship to various parameters including tumor stage, histopathologic characteristics, and disease outcomes. High-level HMGA1 expression predicted poor survival for patients with stage II tumors (S2A Fig). HMGA1 staining was also more intense in stage II tumors (S2C and S2D Fig) and in tumors with perineural invasion (S2E and S2F Fig). Interestingly, expression data from TCGA mRNA-seq studies [37] indicated that high-level expression of HMGA2 correlates inversely with survival (S2B Fig). In tissue arrays HMGA2 expression was greater in non-mucinous tumors (S2G and S2H Fig) and in stage III tumors (S2I and S2J Fig). In aggregate, these data suggest that HMGA1 and HMGA2 are expressed in non-overlapping tumor types, but are both associated with more aggressive phenotypes, and perhaps reduced patient survival.

### HMGA2 Drives a Stem Cell Phenotype and Is Required for Lin28b-Mediated Tumorigenesis

**Fig 6:**
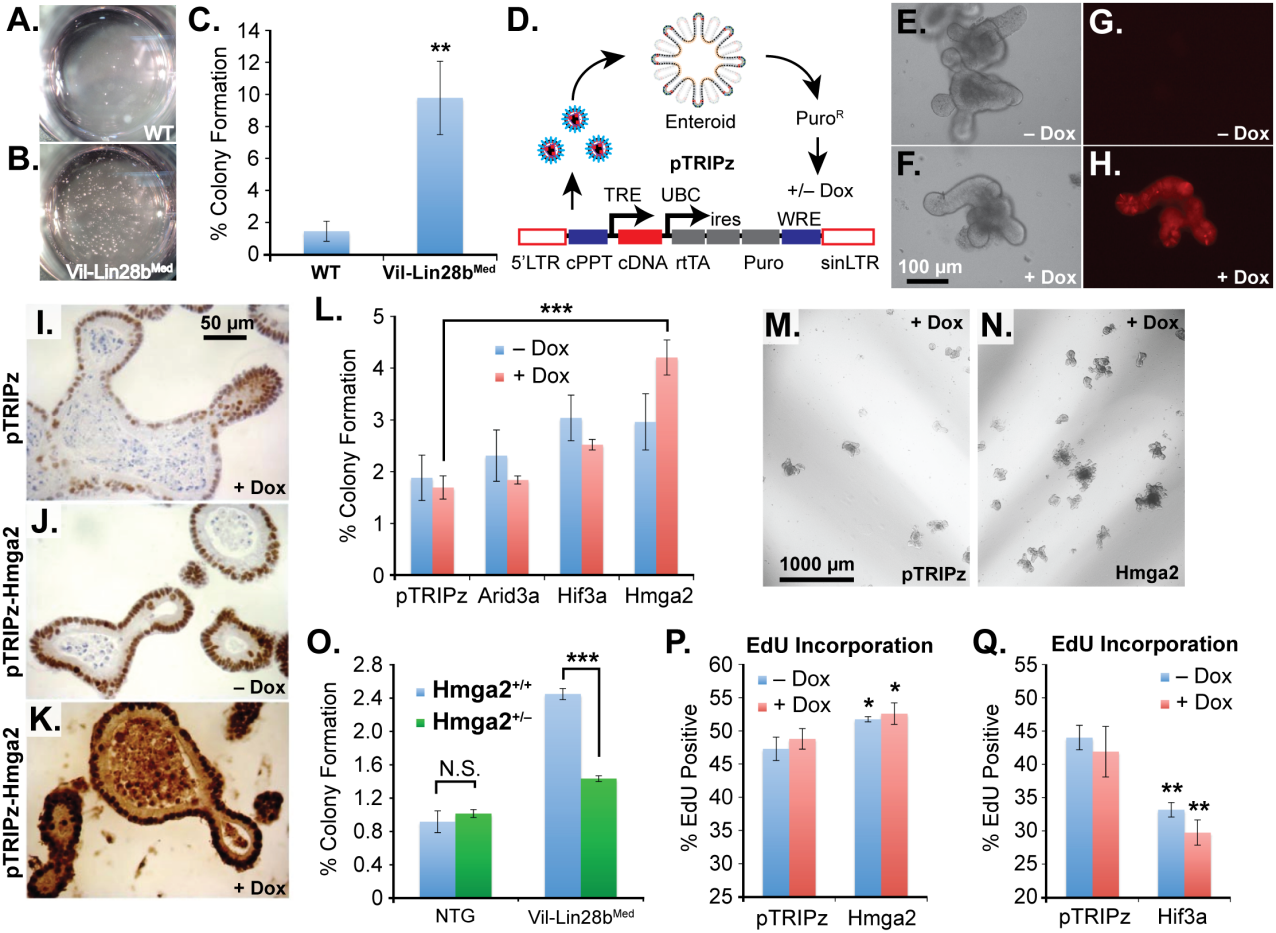
Hmga2 mediates Lin28b effects on stem cell colony formation and enteroid proliferation. Colony formation assay of small intestine enteroids from wild-type (WT) mice (A) and *Vil-Lin28b* ^*Med*^ mice (B). Two wells of a 24-well plate are pictured. C) Quantification of colony forming assay of WT mice and *Vil-Lin28b* ^*Med*^ mice. D) Schematic of lentiviral vector-mediated transduction of enteroids with a “tet-on” doxycycline (dox)-inducible vector. Inducible expression of a turbo RFP reporter in the unmodified pTRIPz vector is readily observed in enteroids (H) vs. untreated cells (G). Phase contrast images of untreated and doxycycline treated enteroids are pictured in (E) and (F), respectively. Immunostaining for Hmga2 in sectioned enteroids from pTRIPz-transduced (I), pTRIPz-Hmga2-transduced (J), and pTRIPz-Hmga2-transduced plus doxycycline (K). L) Comparison of colony forming potential of cells from dissociated enteroids transduced with pTRIPz (empty), Arid3a, Hif3a, or Hmga2 vectors. Phase contrast images of colony formation assays from enteroids transduced with pTRIPz (empty) (M) or Hmga2 (N) vectors. O) Colony forming assays of non-transgenic mice (NTG) or *Vil-Lin28b* ^*Med*^ mice with and without inactivation of one conditional Hmga2 allele using *Vil-Cre*. EdU incorporation of enteroids, as assayed by flow cytometry, transduced with pTRIPz-Hmga2 (P) or pTRIPz-Hif3a (Q) vectors. Cultures were treated with 100 ng/ml doxycycline in all experiments except in (F) and (H), which were treated with 500 ng/ml doxycycline. All experiments were performed at least in triplicate. Student’s two-tailed T-tests were performed to determine significance with * p-value < 0.05, ** p-value < 0.01, and *** p-value < 0.001, relative to pTRIPz vector controls, unless noted otherwise.

We next pursued 3-D culture and manipulation of intestinal organoids (enteroids) to explore the relationship between Let-7 targets and a stem cell phenotype. This method has facilitated the examination of stem cell phenotypes in the intestinal epithelium in multiple studies [34,38–44]. For these experiments we derived enteroids from *Vil-Lin28b* ^*Med*^ mice [18]. We have previously shown that crypt hyperplasia and Hmga2 expression is dependent on Let-7 depletion in crypts from *Vil-Lin28b* ^*Med*^ mice [18]. Enteroids derived from *Vil-Lin28b* ^*Med*^ mice exhibited enhanced colony forming potential of single cells (Fig 6A, 6B and 6C). This is unlikely to be a feature secondary to enhanced stem cell potential conferred by increased association with Paneth cells, as described previously [34], since this cell type is severely depleted following Let-7 repression [18]. To assay exogenous expression of Let-7 targets in enteroids, we used a lentivirus vector for transduction of wild-type mouse small intestine enteroids (Fig 6D–6G). This vector system yields low (Fig 6J) or high-level (Fig 6K) expression, in a doxycycline-dependent manner. We generated stable enteroid lines for inducible expression of mouse *Hmga2*, *Igf2bp2*, *E2F5*, *Arid3a*, or *Hif3a* and assayed colony forming potential and EdU incorporation. We focused on *Hmga2*, rather than *Hmga1*, as it is consistently up-regulated in non-malignant intestinal tissue from *Vil-Lin28b* ^*Med*^ and *Lin28b* ^*Lo*^/*Let7* ^*IEC-KO*^ and thus appears highly dependent on Let-7 [18]. For colony formation, only *Hmga2* over-expression (O/E) exhibited a significant effect, with enhanced formation of new enteroids from singly plated cells (Fig 6L, 6M and 6N), whereas *Igf2bp2*, *E2F5*, *Arid3a*, and *Hif3a* had no apparent effect (Fig 6L and S4A Fig). Expression of *Hmga2*, *Arid3a*, *Hif3a*, or *Igf2bp2* via lentiviral vectors did not induce any change in stem cell markers (S4B–S4K Fig). To determine if Hmga2 was necessary for the enhanced colony formation in *Vil-Lin28b* enteroids, we crossed *Vil-Lin28b* ^*Med*^ mice onto background in which one allele of *Hmga2* is inactivated specifically in the intestine (*Vil-Cre* *^+^* */Hmga2* *^CK/+^*) [45], and generated enteroids. We used *Vil-Lin28b* ^*Med*^ mice because their phenotype appears Let-7-dependent [18] and for simpler breeding. Effects on colony formation by Lin28b were greatly blunted by inactivation of a single *Hmga2* allele (Fig 6O). Hmga2 could also trigger increased EdU incorporation in intestinal enteroids, whereas *Hif3a* repressed it, suggesting opposing effects of *Hmga2* and *Hif3a* on cellular proliferation (Fig 6P and 6Q). Lentiviral-mediated expression and manipulation of the Hmga2 conditional allele were restricted to coding sequence only [45]. Perhaps consistent with its association with a stem cell phenotype, *HMGA2* is also frequently co-expressed with the stem cell markers *MSI1* and *LGR5* in human CRC, and notably, more frequently than any of the other Let-7 targets evaluated here in this study (Fig 5L and S2 Table). Lastly, to evaluate the role of Hmga2 in intestinal tumorigenesis in the context of Let-7 depletion we examined tumor burden in *Vil-Lin28b* ^*Med*^ and *Vil-Lin28b* ^*Med*^ */Hmga2* ^*+/IEC-KO*^ mice. As mentioned earlier, *Vil-Lin28b* ^*Med*^ mice have a lower penetrance of intestinal tumorigenesis compared to *Lin28b* ^*Lo*^/*Let7* ^*IEC-KO*^ mice, with about 50% of animals developing tumors by 9 months of age (S3 Table). Inactivation of one allele of Hmga2 in the intestinal epithelium significantly reduced disease penetrance and tumor burden in *Vil-Lin28b* ^*Med*^ */Hmga2* ^*+/IEC-KO*^ mice (S3 Table).

## Discussion

We have achieved comprehensive depletion of all Let-7 miRNAs in the intestinal epithelium and demonstrated the critical nature of their cumulative tumor-suppressive properties. These effects appear to be due to Let-7, although LIN28B can bind mRNAs and modulate protein levels of targets in the intestinal epithelium [18]. However, this appears unlikely in *Lin28b* ^*Lo*^/*Let7* ^*IEC-KO*^ mice since LIN28B did not have any effect on RNA or protein levels of targets in the context of low-level expression in *Vil-Lin28b* ^*Lo*^ mice [18]. While tumors from *Lin28b* ^*Lo*^/*Let7* ^*IEC-KO*^ mice appear to be more advanced than those from *Vil-Lin28b* ^*Med*^ mice [18], surprisingly there is not a significant difference in tumor multiplicity. Nascent tumorigenesis beginning with aberrant crypt foci and/or microadenomas may occur spontaneously in our mouse model of Let-7 depletion, likely due to sporadic deregulation of Wnt signaling or potential spontaneous loss of other tumor suppressive mechanisms. Therefore, Let-7 may not have the “gatekeeper” function that is characteristic of tumor suppressors such as *APC*. Despite this, there is a link between LIN28B expression in human colon cancer samples and aggressive disease in early stages, which may reflect a role for LIN28B in early neoplastic growth [15]. Supporting this hypothesis is the documentation that LIN28 proteins and Let-7 miRNAs do indeed affect proliferation, migration, and invasion in cell culture models and xenografts of various malignancies [16,17,46–49]. However, the differences between Let-7 target mRNAs in each of these models can be quite disparate; e.g. *KRAS* has a larger effect on tumorigenesis than does *HMGA2* in a non-small cell lung cancer model [49], whereas *HMGA2* appears to have a much larger role in other cancer models [28,50–53], likely as a modifier of chromatin structure and gene expression [54–57].

As documented in developmental programs in *C*. *elegans* and in human cancers, Let-7 miRNAs repress a stem cell phenotype and tumor-initiating phenotype [3], an association we observe here as well. The connection between HMGA2 and a stem cell phenotype in the intestinal epithelium is also intriguing. HMGA2 promotes somatic stem cell specification, with such roles in neural stem cells and hematopoietic stem cells [25–27]. In some contexts, HMGA2 can enhance Wnt signaling, a known driver of the crypt-base-columnar (CBC) intestinal epithelial stem cell phenotype [34,58,59]. This was observed in a mouse model of prostatic intraepithelial neoplasia, where overexpression of Hmga2 in cancer-associated fibroblasts enhances expression of the Wnt ligands *Wnt2*, *Wnt4*, and *Wnt9b*, concomitant with enhanced Wnt signaling and nuclear β-catenin in adjacent neoplastic epithelium [60]. Wnt signaling is required for the enhanced prostatic intraepithelial tumorigenesis induced by Hmga2 in this model [60]. However, we do not observe any effects on Wnt target genes or β-catenin localization in non-malignant *Lin28b* ^*Lo*^/*Let7* ^*IEC-KO*^ intestine tissue, suggesting that Wnt deregulation may be an independent event. A recent study that largely replicated our earlier work found that tumors triggered by transgenic LIN28B expression in the mouse small intestine frequently have mutations in *Ctnnb1* (β-catenin), but not *Apc* [61]. Although the level of induced LIN28B expression in this study is likely much higher than in *Lin28b* ^*Lo*^/*Let7* ^*IEC-KO*^ mice, and therefore different, we suspect that derangements of the Wnt pathway, e.g. in *Ctnnb1*, are also occurring in tumors in *Lin28b* ^*Lo*^/*Let7* ^*IEC-KO*^ mice, as evidenced by frequent nuclear β-catenin.

Alternatively, the co-localization of nuclear β-catenin with intense Hmga2 staining in mouse tumors (Fig 6K–6N) could reflect Wnt signaling enhancement of Hmga2 expression, a phenomenon observed in triple-negative breast cancer, a subtype that also tends to express high LIN28B levels [9,62]. Curiously, in our genetic manipulations of enteroids, exogenous Hmga2 does not affect expression of stem cell markers (such as those assayed in Fig 6I) in transduced intestinal enteroids (S4 Fig). Alternatively, increased Hmga2 expression may enhance survival of stem cells or facilitate the recruitment of a facultative population (such as the quiescent “+4” secretory progenitor stem cell) and entry into the cell cycle. Or, Hmga2 may synergize with deregulated Wnt signaling in the promotion of a stem cell phenotype, which could account for the dramatic up-regulation of stem cell markers we see in tumors from *Lin28b* ^*Lo*^/*Let7* ^*IEC-KO*^ mice. Others have also reported that HMGA2 expression is predictive of aggressive disease and poor outcomes in CRC [63], as similarly found in other cancers [50].

While HMGA2 is playing a key role, it is likely that the effects of Let-7 on an intestinal stem cell phenotype and epithelial tumorigenesis are dependent on the collective and/or cooperative role of multiple Let-7 targets. Not uncommonly, additive roles of target genes are uncovered in the genetic dissection of a single pathway, such as that seen for PDGF-receptor signaling and the collective biological contribution of multiple target genes dependent on *Pdgfra* and *Pdgfrb* [64]. However, it is challenging to dissect the combinatorial relationships among a dozen candidate targets, especially in mouse models. An oncogenic function of *HMGA1* and *IGF2BP1* has been reported in other cancers, including colon cancer, with evidence that both factors enhance tumorigenesis [65,66]. Dissecting the interaction and possible cooperation of Let-7 target mRNAs is critical for designing strategies to ameliorate the loss of Let-7 in human cancers via combinatorial targeted therapies against multiple oncogenes.

## Materials and Methods

### Ethics Statement

Mouse studies were approved by the University of Pennsylvania Animal Care and Use Committee, protocol #802791.

### Mouse Models

*Vil-Lin28b* ^*Lo*^, *Vil-Lin28b* ^*Med*^, and Let7^IEC-KO^ mice were described previously [18], and were maintained via backcrosses to C57BL/6J. *Vil-Lin28b* ^*Med*^ mice express Lin28b protein approximately 2-fold higher than *Vil-Lin28b* ^*Lo*^ mice. To obtain *Lin28b* ^*Lo*^/*Let7* ^*IEC-KO*^ mice, *VilCre* ^*+*^ */Let7* ^*lox/lox*^ mice were mated with *Vil-Lin28b* ^*Lo*^/*Let7* ^*lox/lox*^ mice to get *Vil-Lin28b* ^*Lo*^/*VilCre* ^*+*^/*Let7* ^*lox/lox*^, and all other possible genotypes. *Let7* ^*lox/lox*^ mice were considered wild-type and possess all Let-7 miRNAs at levels insignificantly different from wild-type mice [18]. Conditional null *Hmga2^Ck^* mice were described previously [45]. Mice were sacrificed at 12 weeks or between 10 and 14 months of age for dissection, isolation of tissues for histology and immunohistochemistry, and isolation of intestinal epithelia. Pathological criteria for mouse intestinal tumors were used as previously defined [67]. Intestinal adenomas are exophytic growths without evidence of invasion characterized by enlarged variably hyperchromatic nuclei with altered glandular architecture, including enlarged crypts and budding, irregular glands. For purposes of defining an adenocarcinoma, tumor invasion through the lamina propria into the muscularis mucosa and eventually beyond must clearly be seen.

### Quantitative RT-PCR

For mRNA expression analysis of mouse tissue, whole jejunum, total intestinal epithelium, or crypt epithelium was isolated for homogenization in Trizol (Life Technologies). Total epithelium or crypts were isolated as described previously [18]. Total RNA (2–5 μg) was used for cDNA synthesis with oligo dT primers and Superscript III RT (Life Technologies) according to the manufacturer instructions. QPCR was performed using Taqman technology or Sybr green using the TaqMan Fast Universal PCR Master Mix (2X), no AmpErase UNG (Life Technologies) or the Power SYBR Green PCR Master Mix (Life Technologies). Expression levels of queried mRNAs were normalized to β-actin (*Actb*) and *Hprt* or *Gapdh* mRNA levels. Let-7 miRNAs were quantified using Taqman Q-RT-PCR kits (Life Technologies), according to the manufacturers instructions and normalized to U6 and SNO135 small RNA levels. Primers and Taqman probes are listed in S3 Table.

Human colon cancer tumor specimens, along with adjacent matched non-malignant tissue, were obtained from the Siteman Cancer Center Tissue Procurement Core as fresh frozen sections. Twenty samples (11 pairs) were obtained and total RNA was isolated following homogenization with Trizol (Life Technologies). For qualitative evaluation of RNA integrity 2 μg of total RNA was electrophoresed on a 1% agarose gel. For evaluation of mRNAs, 1 μg of total RNA was used for reverse transcriptase using the iScript reverse transcriptase kit (BioRad), while miRNAs were quantified using Taqman Q-RT-PCR kits (Life Technologies), according to the manufacturers instructions. Levels of mRNAs were assayed using standard primer pairs and SsoFast EvaGreen Supermix (Biorad) and normalized to cyclophilin-A (*PPIA*) and β-2 microglobulin (*B2M*). Let-7a and Let-7b miRNAs were normalized to U6 and RNU6B RNAs.

### Enteroid/Tumoroid Culture

Crypts were isolated as described previously [18] and cultured in EGF, Noggin, and Rspo1 (ENR) medium [34]. Before plating, crypts were counted and re-suspended in a mixture of 80% Matrigel (BD Biosciences) and 20% ENR at a concentration of 20 crypts per μl. For initial plating and the first three days of culture, crypts were grown in the presence of 10 μM Rho kinase (ROCK) inhibitor (Y27632). Medium was then changed every 3 days with fresh ENR medium. For lentiviral transduction the pTRIPz vector was modified for expression of mouse Hmga2 (NM_010441.2), E2f5 (NM_007892.2), Igf2bp2 (NM_183029.2), Arid3a (NM_001288625.1) or Hif3a (NM_016868.3) by cloning the open reading frames from these cDNAs between the *Age*I and *Mlu*I sites within pTRIPz. Enteroids were transduced with lentiviral particles as described previously and selected with 2 μg/ml puromycin [68].

### Colony Formation Assays

Enteroids were mechanically dissociated by pipetting up and down in 4 ml basal medium and centrifuged at 100 x g for 2 min. Enteroids were then re-suspended in 0.5 ml TrypLE Express (Life Technologies) containing 250 U/ml DNase I (1:200) and 10 μM ROCK inhibitor. Enteroids were incubated 5 minutes at 37°C with periodic vortexing every 60 sec. To this we added one volume basal medium with 5% FBS (with DNase and ROCKi) and spun 5 min at 200 x g. Cells were re-suspended in 0.5 ml pre-warmed basal medium with 50 U/ml DNase I and 10 μM ROCK inhibitor and incubated 5 minutes at room temperature with periodic vortexing. Single cells were then plated in triplicate at a concentration of 2500 cells per cm^2^ in 80% Matrigel, 20% ENR, and over-layed with ENR medium plus 10 μM ROCK inhibitor. Colonies of growing, budding enteroids were counted 5–7 days after plating.

For assaying 5-ethynyl-2´-deoxyuridine (EdU) incorporation, enteroids were given fresh new medium containing 10 μM EdU and incubated for 2 hours. Enteroids were then isolated as performed above to obtain a single cell suspension, then fixed for Click-iT labeling and flow cytometry using the Click-iT Plus EdU Alexa Fluor 488 Flow Cytometry Assay Kit (Life Technologies). Fixation and labeling was carried out according to the manufacturer instructions.

### Tumoroid Cultures

Mouse intestinal tumors were isolated for culture by micro-dissecting tumor tissue away from normal adjacent mucosa using a dissecting microscope. Two pieces of tumor were processed for RNA isolation and histology and immunohistochemistry while a third piece was dissociated for culture. For tumoroid culture, tumors were placed into HBSS containing 10 mM EDTA, 1 mM N-acetyl-cysteine (NAC), and 10 μM ROCK inhibitor (Y27632) and incubated at 37°C with periodic vortexing for approximately 5 to 10 minutes until the epithelium began to detach. Isolated epithelium was then washed three times with sterile basal medium and plated in culture as done above for enteroids in ENR medium. After 1–2 weeks of continuous culture and 1–2 passages, tumoroids were placed into medium lacking Noggin and Rspo1. While most enteroids died, small rare surviving colonies could be observed after 3–5 days of culture. These tumoroid cysts were maintained in medium lacking Noggin and Rspo1.

### Immunohistochemistry (IHC)

Paraffin-embedded enteroids, intestinal tissue, and tissue microarrays were incubated at 56°C prior to de-waxing and rehydration. Antigens were retrieved by boiling sections in 10 mM citric acid, pH 6.0, for 2 hrs. Samples were blocked in 1% BSA, 0.3% Triton-X-100, and 10% normal goat serum for 1 hr. Endogenous peroxidases were quenched in 3% hydrogen peroxide for 5 minutes. In conjunction with biotin-conjugated secondary antibodies (Jackson ImmunoResearch, diluted 1:200) stains were developed with the VECTASTAIN Elite ABC Kit (Vector Laboratories, cat# PK-6100) and the DAB Peroxidase (HRP) Substrate Kit (Vector Laboratories, cat# SK-4100). Tissues were dehydrated and cover-slipped with Cytoseal (Thermo Scientific, cat# 8310–4). Primary antibodies used for IHC were anti-ARID3A (1:100, ProteinTech, Chicago IL, cat# 14068-1-AP), anti-β-catenin [D10A8] XP Rabbit mAb (1:100, Cell Signaling, Danvers MA, cat# 8480), rabbit anti-HIF3A antibody (1:200, Sigma-Aldrich, St. Louis MO, cat# SAB2900410), anti-HMGA1 antibody [EPR7839] (1:250, Abcam, Cambridge MA, cat# ab129153), and anti-HMGA2 [D1A7] rabbit mAb (1:400, Cell Signaling, Danvers MA, cat# 8179).

### Human Cancer Dataset Analyses

We examined gene expression in CRC specimens from a cohort of 416 CRC patients from a TCGA dataset using the cancer genome browser at UCSC (https://genome-cancer.ucsc.edu/proj/site/hgHeatmap/) (Cline MS 2013; Lopez-Bigas N 2013; Goldman M 2012; Sanborn JZ 2011; Vaske CJ 2010; Zhu J 2009). For examination of Let-7 miRNA expression and expression relative to candidate target genes we examined a cohort of 199 CRC patients from the TCGA Pan-Cancer analysis project visualized using the starbase miRNA CLIP-seq portal (http://starbase.sysu.edu.cn/) (Li JH et al., Nucleic Acids Res. 2014; Yang JH et al., Nucleic Acids Res. 2011).

## Supporting Information

S1 Fig Let-7 miRNAs and Let-7 Target anti-correlation in CRC TCGA datasets. A-C) Box-and-whisker plots for Let-7a, Let-7b, and Let-7c, demonstrating significant down-regulation in colon and rectal cancer (CRC) miRNA-seq dataset. Box plot whiskers represent 1.5x the interquartile range (IQR) above the third quartile or below the first quartile. D-I) Scatter plots of Let-7 miRNA expression vs. target mRNA levels from CRC miRNA-seq and mRNA-seq datasets. Pearson correlation coefficients and p-values are indicated on each graph.
(TIF)

S2 Fig HMGA1 and HMGA2 tumor tissue array analysis. A) Kaplan-Meier curve depicting survival in patients with high HMGA1 staining scores vs. low staining scores. B) Kaplan-Meier curve depicting survival in patients with high HMGA2 levels from RNA-seq data [37]. High levels are defined as expression at least one standard deviation above the mean. C) Contingency table of HMGA1 staining intensity (high or low) vs. tumor stage (II or III). D) Chi-Square test for data in (C) revealing a significant difference in staining intensity, with more stage II tumors exhibiting higher levels of HMGA1 staining. E) Contingency table of HMGA1 staining intensity (high or low) vs. tumor perineural invasion. F) Chi-Square test for data in (E) revealing a significant difference in staining intensity, with more tumors with perineural invasion exhibiting higher levels of HMGA1 staining. G) Contingency table of HMGA2 staining intensity (high or low) vs. tumor mucinous phenotype. H) Chi-Square test for data in (G) revealing a significant difference in staining intensity, with non-mucinous tumors exhibiting higher levels of HMGA2 staining. I) Contingency table of HMGA2 staining score (high/low) vs. tumor stage (I, II, or III). J) Chi-Square test for data in (I) revealing a significant difference in staining intensity, with stage III tumors exhibiting higher levels of HMGA2.
(TIF)

S3 Fig Arid3a protein is unchanged while Hif3a protein is decreased in invasive areas of adenocarcinomas. Immunohistochemical staining for Arid3a (A-D) and Hif3a (E-H), in sections from WT small intestine (S.I.) (A, E), *Lin28b* ^*Lo*^ */Let7* ^*IEC-KO*^ S.I. (B, F), *Lin28b* ^*Lo*^ */Let7* ^*IEC-KO*^ adenoma (C, G), and *Lin28b* ^*Lo*^ */Let7* ^*IEC-KO*^ adenocarcinoma (D, H). All pictures are at same magnification, with scale bar = 100 μm.
(TIF)

S4 Fig E2f5 and Imp2 do not significantly affect colony forming potential while Arid3a, Hif3a, Hmga2, and Igf2bp2 (Imp2) do not significantly affect stem cell marker expression. A) Colony forming assay in WT mouse small intestine enteroids transduced with pTRIPz (empty vector), pTRIPz-E2f5, or pTRIPz-Imp2 (Igf2bp2). Enteroids were treated with 100 ng/ml doxycycline for 5 days, then dissociated into single cells. B-K) Q-RT-PCR for epithelial stem cell markers in WT mouse small intestine enteroids transduced with pTRIPz vectors expressing Arid3a, Hif3a, Hmga2, or Imp2 (Igf2bp2) and treated for 5 days with 100 ng/ml doxycycline. Assays were performed in triplicate. Q-RT-PCR values in B-D were normalized to Gapdh (*Gapdh*) and β-Actin (*Actb*).
(TIF)

S1 Table Polyp and adenocarcinoma incidence in *WT*, *Vil-Lin28b* ^*Lo*^, *Let7* ^*IEC-KO*^, *Lin28b* ^*Lo*^ */Let7* ^*IEC-KO*^ mice. (PDF)

S2 Table Co-expression of Let-7 Targets HMGA2, ARID3A, IGF2BP2, PLAGL2, HMGA1, HIF3A, E2F5, NR6A1, MYCN, and DDX19A with stem cell markers (LGR5, EPHB2, ASCL2, MSI1, z-score threshold +/– = 1) in two human colon cancer datasets from TCGA (http://www.cbioportal.org/) [37,69,70]. IGF2BP1 did not correlate with any stem cell markers.
(PDF)

S3 Table Tumor incidence in *Vil-Lin28b* ^*Med*^, and *Vil-Lin28b* ^*Med*^ */Hmga2* ^*+/IEC-KO*^ mice sacrificed at 9 months of age. (PDF)

S4 Table Primers used for RT-PCR. (PDF)

S1 Methods Supplemental experimental procedures. (DOCX)
