# Supplementary figures and images for: Let-7 Represses Carcinogenesis and a Stem Cell Phenotype in the Intestine via Regulation of Hmga2
Source: PLoS Genet. 2015 Aug 5;11(8):e1005408. doi: 10.1371/journal.pgen.1005408 (PMC4526516; doi:10.1371/journal.pgen.1005408)

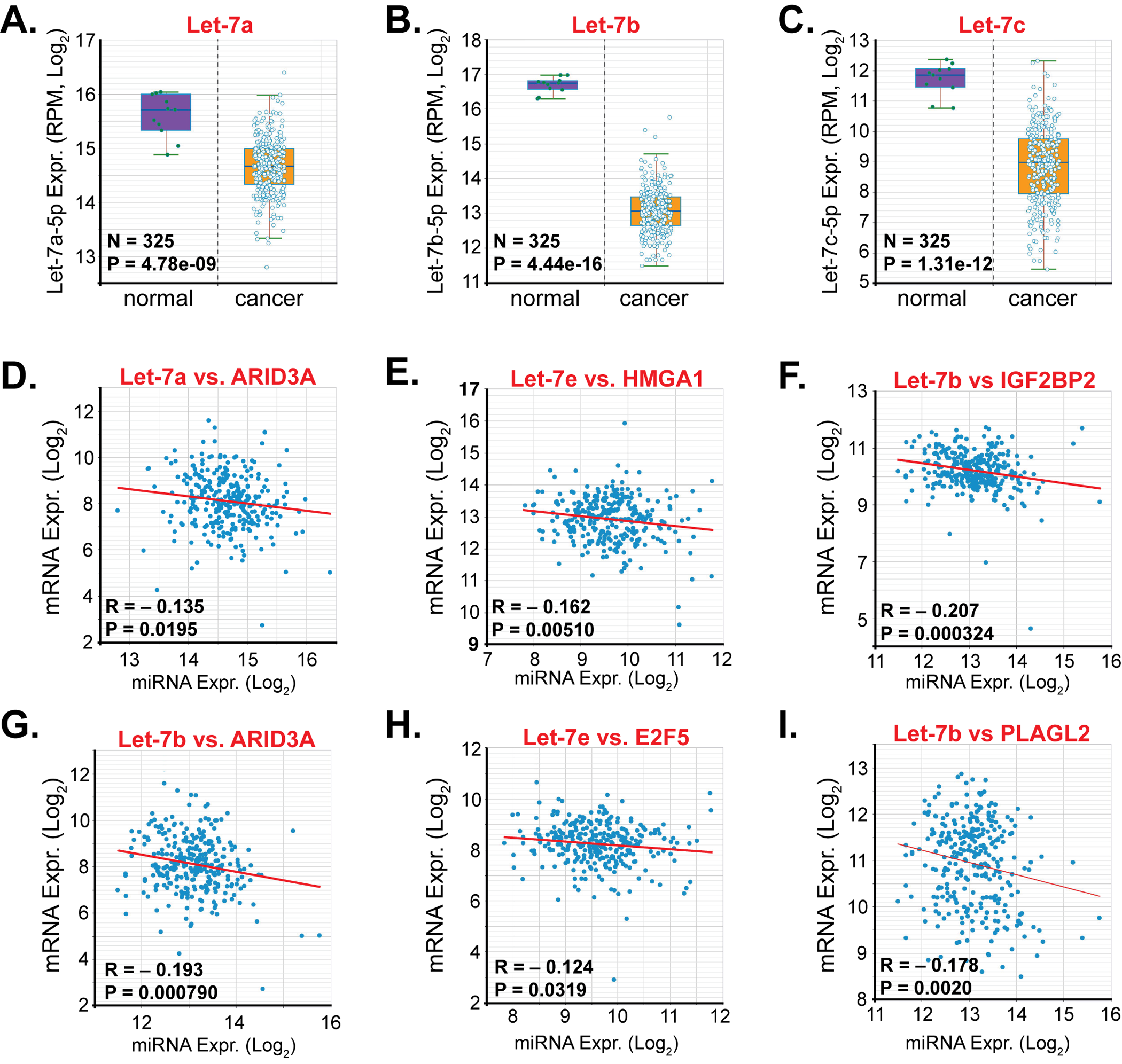

Supplement: S1 Fig — A-C) Box-and-whisker plots for Let-7a, Let-7b, and Let-7c, demonstrating significant down-regulation in colon and rectal cancer (CRC) miRNA-seq dataset. Box plot whiskers represent 1.5x the interquartile range (IQR) above the third quartile or below the first quartile. D-I) Scatter plots of Let-7 miRNA expression vs. target mRNA levels from CRC miRNA-seq and mRNA-seq datasets. Pearson correlation coefficients and p-values are indicated on each graph. (TIF) [file pgen.1005408.s001.tif]

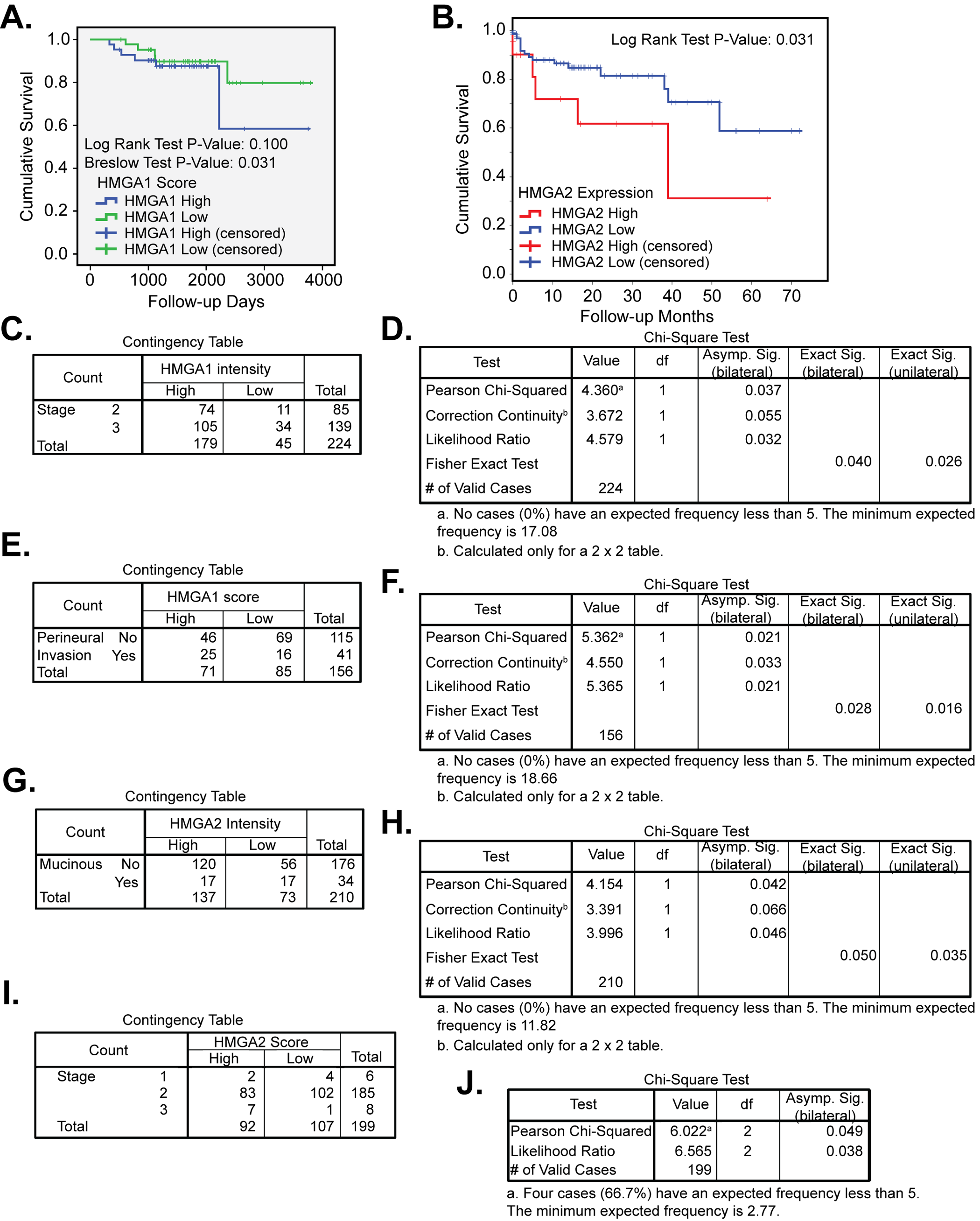

Supplement: S2 Fig — A) Kaplan-Meier curve depicting survival in patients with high HMGA1 staining scores vs. low staining scores. B) Kaplan-Meier curve depicting survival in patients with high HMGA2 levels from RNA-seq data [37]. High levels are defined as expression at least one standard deviation above the mean. C) Contingency table of HMGA1 staining intensity (high or low) vs. tumor stage (II or III). D) Chi-Square test for data in (C) revealing a significant difference in staining intensity, with more stage II tumors exhibiting higher levels of HMGA1 staining. E) Contingency table of HMGA1 staining intensity (high or low) vs. tumor perineural invasion. F) Chi-Square test for data in (E) revealing a significant difference in staining intensity, with more tumors with perineural invasion exhibiting higher levels of HMGA1 staining. G) Contingency table of HMGA2 staining intensity (high or low) vs. tumor mucinous phenotype. H) Chi-Square test for data in (G) revealing a significant difference in staining intensity, with non-mucinous tumors exhibiting higher levels of HMGA2 staining. I) Contingency table of HMGA2 staining score (high/low) vs. tumor stage (I, II, or III). J) Chi-Square test for data in (I) revealing a significant difference in staining intensity, with stage III tumors exhibiting higher levels of HMGA2. (TIF) [file pgen.1005408.s002.tif]

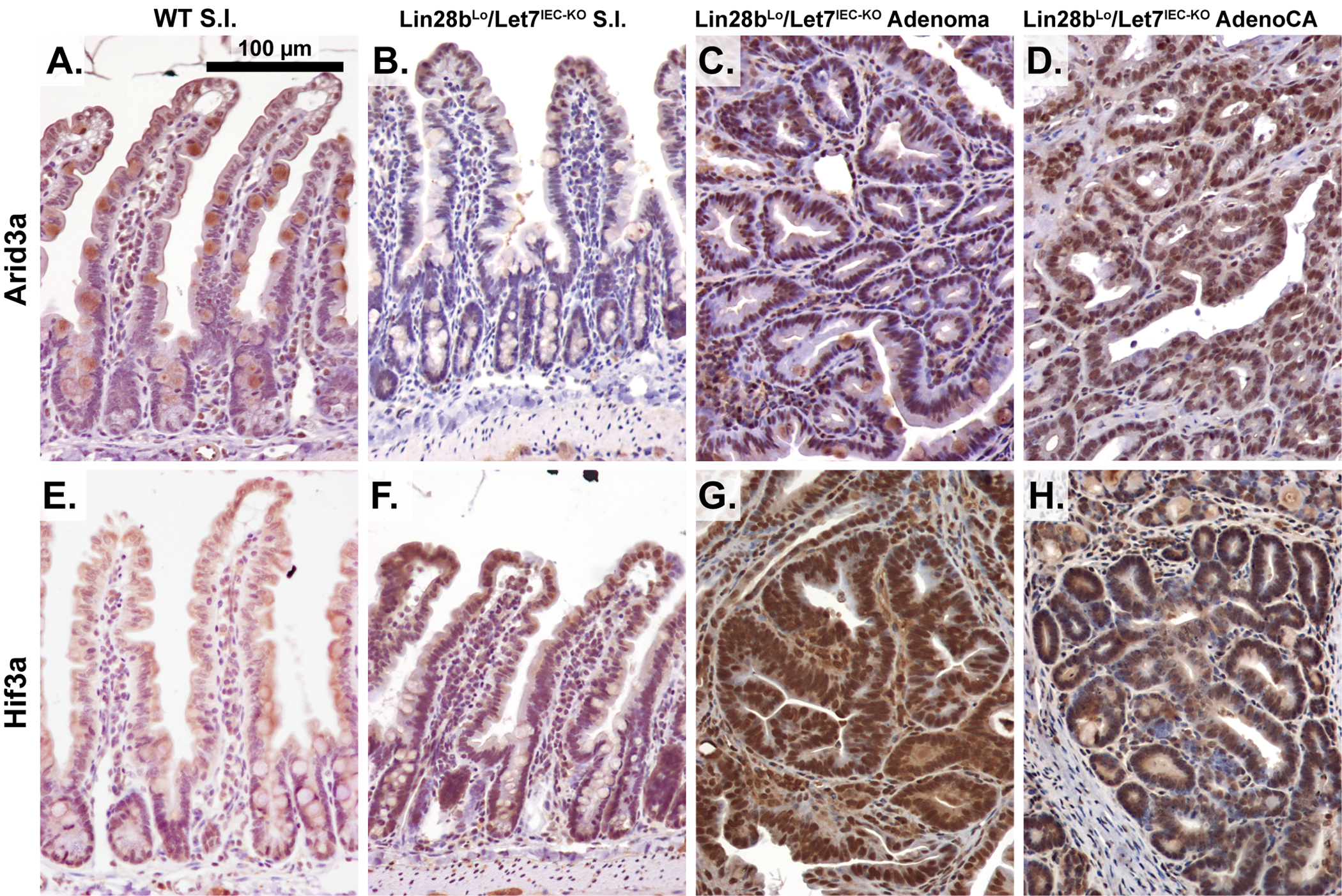

Supplement: S3 Fig — Immunohistochemical staining for Arid3a (A-D) and Hif3a (E-H), in sections from WT small intestine (S.I.) (A, E), Lin28b Lo /Let7 IEC-KO S.I. (B, F), Lin28b Lo /Let7 IEC-KO adenoma (C, G), and Lin28b Lo /Let7 IEC-KO adenocarcinoma (D, H). All pictures are at same magnification, with scale bar = 100 μm. (TIF) [file pgen.1005408.s003.tif]

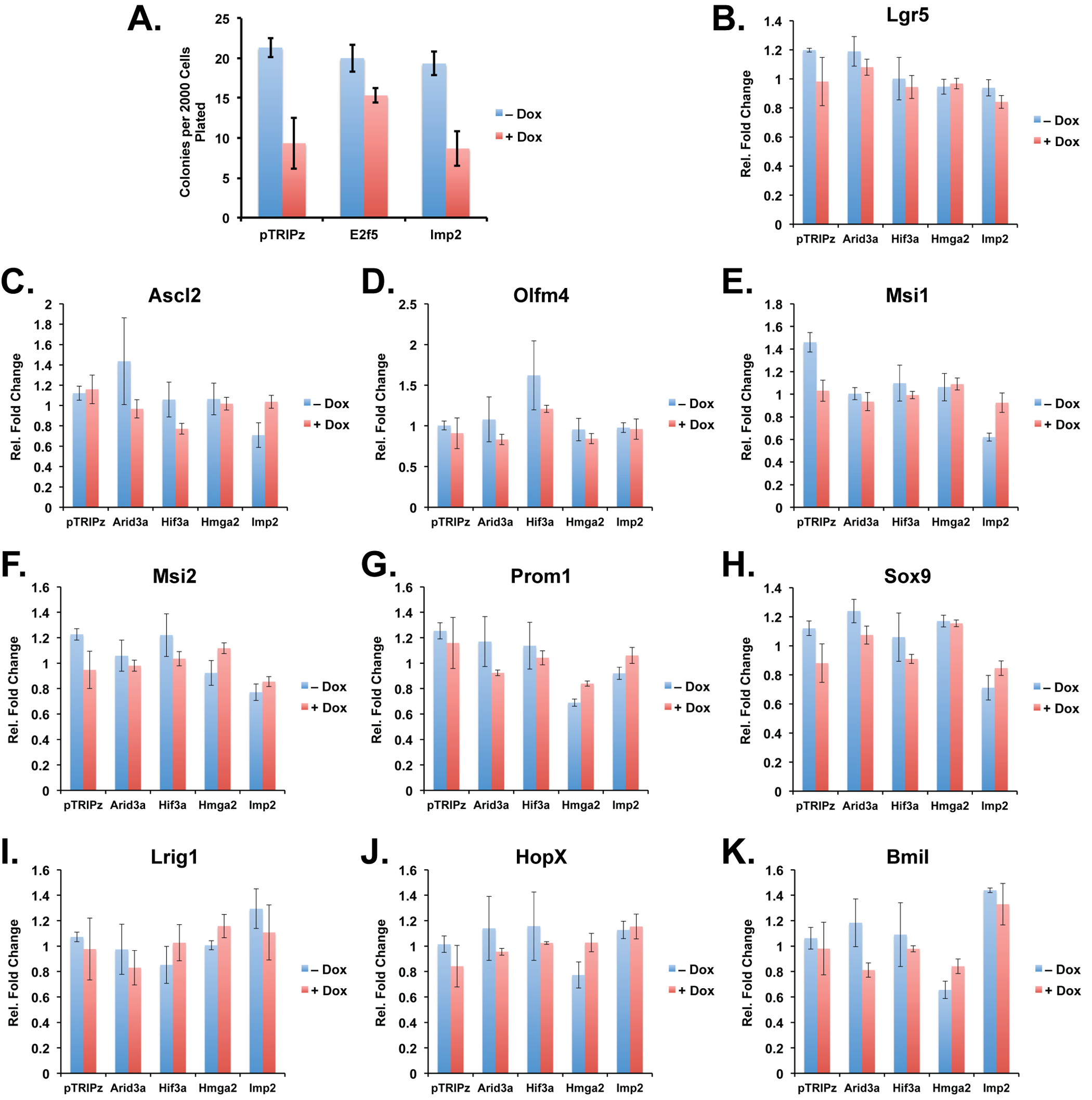

Supplement: S4 Fig — A) Colony forming assay in WT mouse small intestine enteroids transduced with pTRIPz (empty vector), pTRIPz-E2f5, or pTRIPz-Imp2 (Igf2bp2). Enteroids were treated with 100 ng/ml doxycycline for 5 days, then dissociated into single cells. B-K) Q-RT-PCR for epithelial stem cell markers in WT mouse small intestine enteroids transduced with pTRIPz vectors expressing Arid3a, Hif3a, Hmga2, or Imp2 (Igf2bp2) and treated for 5 days with 100 ng/ml doxycycline. Assays were performed in triplicate. Q-RT-PCR values in B-D were normalized to Gapdh (Gapdh) and β-Actin (Actb). (TIF) [file pgen.1005408.s004.tif]
