# Supplementary material for: Let-7 Represses Carcinogenesis and a Stem Cell Phenotype in the Intestine via Regulation of Hmga2
Source: PLoS Genet. 2015 Aug 5;11(8):e1005408. doi: 10.1371/journal.pgen.1005408 (PMC4526516; doi:10.1371/journal.pgen.1005408)
Supplement: S1 Table — (PDF) [file pgen.1005408.s005.pdf]

**Genotype**	**Mice w/Tumor (%)**	**Mice w/AdenoCA (%)**	**Avg # Tumors Per Affected Mouse**	**Avg # of Tumors Classified as AdenoCA**
WT	0/6	0/6	0	0
Vil-Lin28b^{Lo}	1/9 (11%)	0/9	1	0
Let7^{IEC-KO}	1/16 (6%)	0/16	1	0
Lin28b^{Lo}/ Let7^{IEC-KO}	7/7 (100%)*<br>*p = 0.0006 by Fisher Exact test	6/7 (86%)**<br>**p = 0.005 by Fisher Exact test	2.86<br>SEM = 0.508	1.14 (40%)<br>SEM = 0.261
