# Supplementary material for: Let-7 Represses Carcinogenesis and a Stem Cell Phenotype in the Intestine via Regulation of Hmga2
Source: PLoS Genet. 2015 Aug 5;11(8):e1005408. doi: 10.1371/journal.pgen.1005408 (PMC4526516; doi:10.1371/journal.pgen.1005408)
Supplement: S2 Table — IGF2BP1 did not correlate with any stem cell markers. (PDF) [file pgen.1005408.s006.pdf]

Gene A	Gene B	P-value (Fisher exact)	Log of Odds	Dataset	Platform	N =
**HMGA2**	MSI1	0.002	1.432	Ref: [70]	RNA-Seq	244
**HMGA2**	LGR5	0.009	1.317	Ref: [70]	RNA-Seq	244
**HMGA2**	LGR5	0.006	0.812	Ref: [70]	Microarray	224
**HMGA2**	LGR5	0.01	0.784	TCGA Provisional	Microarray	222
**HMGA2**	MSI1	0.016	0.949	TCGA Provisional	RNA-Seq	365
**HMGA2**	LGR5	0.022	0.805	TCGA Provisional	RNA-Seq	365
**ARID3A**	MSI1	0.002	1.699	Ref: [70]	RNA-Seq	244
**ARID3A**	ASCL2	0.004	1.065	TCGA Provisional	Microarray	222
**ARID3A**	ASCL2	0.019	0.847	Ref: [70]	Microarray	224
**ARID3A**	MSI1	0.043	0.793	TCGA Provisional	RNA-Seq	365
**IGF2BP2**	ASCL2	0.014	0.897	TCGA Provisional	Microarray	222
**IGF2BP2**	ASCL2	0.031	0.79	Ref: [70]	Microarray	224
**IGF2BP2**	LGR5	0.046	0.551	TCGA Provisional	Microarray	222
**IGF2BP2**	ASCL2	0.049	0.447	TCGA Provisional	RNA-Seq	365
**PLAGL2**	LGR5	<0.001	1.093	TCGA Provisional	RNA-Seq	365
**PLAGL2**	MSI1	0.004	1.064	TCGA Provisional	RNA-seq	365
**HMGA1**	EPHB2	0.006	1.241	Ref: [70]	RNA-Seq	244
**HMGA1**	ASCL2	0.011	0.749	Ref: [70]	RNA-Seq	244
**HIF3A**	MSI1	0.002	1.625	Ref: [70]	RNA-Seq	244
**HIF3A**	MSI1	0.016	1.145	TCGA Provisional	RNA-Seq	365
**E2F5**	LGR5	0.017	1.012	Ref: [70]	RNA-Seq	244
**E2F5**	LGR5	0.049	0.474	TCGA Provisional	RNA-Seq	365
**NR6A1**	LGR5	0.036	0.913	Ref: [70]	RNA-Seq	244
**NR6A1**	MSI1	0.039	0.622	TCGA Provisional	RNA-Seq	365
**MYCN**	MSI1	0.039	0.747	TCGA Provisional	RNA-Seq	365
**DDX19A**	ASCL2	0.049	0.563	Ref: [70]	Microarray	224
