# Supplementary material for: Let-7 Represses Carcinogenesis and a Stem Cell Phenotype in the Intestine via Regulation of Hmga2
Source: PLoS Genet. 2015 Aug 5;11(8):e1005408. doi: 10.1371/journal.pgen.1005408 (PMC4526516; doi:10.1371/journal.pgen.1005408)
Supplement: S3 Table — (PDF) [file pgen.1005408.s007.pdf]

Genotype	Mice w/Tumor (%)	Avg # Tumors Per Mouse
Vil-Lin28b^{Med}	12/23 (52%)	0.739 (SEM = 0.169)
Vil-Lin28b^{Med}/ Hmga2$^{+/IEC-KO}$	1/9 (11.1%) p = 0.038 by Fisher Exact test (1-tailed)	0.111† (SEM = 0.111) † p = 0.017 by Student's T-test (1-tailed)
