# Supplementary material for: Let-7 Represses Carcinogenesis and a Stem Cell Phenotype in the Intestine via Regulation of Hmga2
Source: PLoS Genet. 2015 Aug 5;11(8):e1005408. doi: 10.1371/journal.pgen.1005408 (PMC4526516; doi:10.1371/journal.pgen.1005408)
Supplement: S4 Table — (PDF) [file pgen.1005408.s008.pdf]

NAME	TARGET	SEQUENCE
mCD44 (F)<br>mCD44 (R)	Mus CD44	(F): TTC ATC CCA ACG CTA TCT GTG<br>(R): CGA AGG AAT TGG GTA GGT CTG
mAxin2 (F)<br>mAxin2 (R)	Mus Axin2	(F): TAG GTT CCG GCT ATG TCT TTG<br>(R): TGT TTC TTA CTC CCC ATG CG
mDdx19a (F)<br>mDdx19a (R)	Mus Ddx19a	(F): GTC AAG TCG ATG AGC AGT TTG<br>(R): CCT CTT CCT CTG TCT TTT CTG C
mMycn (F)<br>mMycn (R)	Mus nMyc	(F): GTC TGT TCC AGC TAC TGC C<br>(R): TCC TCT TCA TCT TCC TCC TCG
mPlagl2 (F)<br>mPlagl2 (R)	Mus Plagl2	(F): GCT TTT GCC TCC AAG TAC AAG<br>(R): TTC CGC AGA TGG TCC TTT C
mTrim6 (F)<br>mTrim6 (F)	Mus Trim6	(F): AGC CAC GGA TTC CAG ATT G<br>(R): GCA GAT AGG GCA GGT TAC TTC
mIgf2bp2 (F)<br>mIgf2bp2 (R)	Mus mIgf2bp2	(F): TCT CGG GTA AAG TGG AAT TGC<br>(R): TGT CCC ATA TTC AGC CAA CAG
mIgf2bp1 (F)<br>mIgf2bp1 (R)	Mus mIgf2bp1	(F): GAG CAG ATG GTA CAA GTG<br>(R): TTG GAG TCA GGT GTT TCT GG
mHmga2 (F)<br>mHmga2 (R)	Mus Hmga2	(F): CAA GAG GCA GAC CTA GGA AAT G<br>(R): GAT CCA ACT GAT GCT GAG GTA G
mHif3a (F)<br>mHif3a (R)	Mus Hif3a	(F): GAG TGA TCC ACG ACT GAA CTG<br>(R): CCA ACC CTT TGT CCT CTG AG
mArid3a (F)<br>mArid3a (R)	Mus Arid3a	(F): CCA GTC ATA TGG CAT CCC AG<br>(R): GTC CGC ATC CAG TTC ATA GAG
mLin28b (F)<br>mLin28b (R)	Mus Lin28b	(F): GAG TCA ATA CGG GTA ACA GGC<br>(R): TTC TCG CAC AGT CCA CATSA
mE2f5 (F)<br>mE2f5 (R)	Mus E2f5	(F): ACC TGA CCG AAG ATA ATG CC<br>(R): ACT GTC TGC TCC TTG AAG TTG
mHmga1 (F)<br>mHmga1 (R)	Mus Hmga1	(F): GGA AAA GGA TGG GAC TGA GAA G<br>(R): TCT TGC TTC CCT TTG GTC G
mZc3hav1 (F)<br>mZc3hav1 (R)	Mus Zc3hav1	(F): CCT ATC AAC GTC CAA GTC CTG<br>(R): GCA GTA GCC ATA AGG GAC ATC G
mCdc34 (F)<br>mCdc34 (R)	Mus Cdc34	(F): CTA CTA TGA GGG CGG CTA CT<br>(R): GGG ATG GAG AAT GGA GAT GC
mWnt3 (F)<br>mWnt3 (F)	Mus Wnt3	(F): AGC TGC CAA GAG TGT ATT CG<br>(R): CTA GAT CCT GCT TCT CAT GGG
mEphb2 (F)<br>mEphb2 (R)	Mus Ephb2	(F): CAG TAC CGG AAA TTC ACC TCG<br>(R): TCT GTA GTC CTG TTC AAT GGC
mMsi1 (F)<br>mMsi1 (R)	Mus Msi1	(F): GTT TCG GCT TCG TCA CTT TC<br>(R): CCA TCT TAG GCT GTG CTC TT
mMsi2 (F)<br>mMsi2 (R)	Mus Msi2	(F): GCG ATG CTG ATG TTC GAC AA<br>(R): TCT CCA CAA CGT CTT CAT TCT CA
mLrig1 (F)<br>mLrig1 (R)	Mus Lrig1	(F): GAA CAC CTG AAC CTT GGA G<br>(R): CTG CAG CAT CCT ACC CAT TAG

mSox9 (F)<br>mSox9 (R)	Mus Sox9	(F): CAA GAC TCT GGG CAA GCT C<br>(R): GGG CTG GTA CTT GTA ATC GG
mBmi1 (F)<br>mBmi1 (R)	Mus Bmi1	(F): CTG GAG AAG AAA TGG CCC ACT A<br>(R): CTC ATC TTC ATT CTT TTG CAA GTT G
mProm1 (F)<br>mProm1 (R)	Mus Prom1	(F): GAA AAG TTG GTC TGC GAA CC<br>(R): TCT CAA GCT GAA AAG CAG CA
mHopx (F)<br>mHopx (R)	Mus Hopx	(F): CGG AGG ACC AGG TGG AGA T<br>(R): CCG GGT GCT TGT TGA CCT T
mAscl2 (F)<br>mAscl2 (R)	Mus Ascl2	(F): GGC TGC TCT GAG CCT ACC T<br>(R): TAG GTC CAC CAG GAG TCA CC
mLgr5 (F)<br>mLgr5 (R)	Mus Lgr5	(F): CAG TGT GGA CGA CCT TCA TAA GAA<br>(R): AAG GTC CCG CTC ATC TTG AAC
mOlfm4 (F)<br>mOlfm4 (R)	Mus Olfm4	(F): TGG CCC TTG GAA GCT GTA GT<br>(R): ACC TCC TTG GCC ATA GCG AA
mHprt (F)<br>mHprt (R)	Mus Hprt	(F): CCC CAA AAT GGT TAA GGT TGC<br>(R): AAC AAA GTC TGG CCT GTA TCC
mGapdh (F)<br>mGapdh (R)	Mus Gapdh	(F): GGG TGT GAA CCA CGA GAA ATA<br>(R): AGT GAT GGC ATG GAC TGT G
mActin (F)<br>mActin (R)	Mus Actin	(F): ACC CAG ATC ATG TTT GAG ACC<br>(R): AGA GCA TAG CCC TCG TAG AT
hEPHB2(F)<br>hEPHB2(R)	Human EPHB2	(F): ATA CCT CAG ACC CCA CCT AC<br>(R): TCA CCT CCC ACA TGA CAA TG
hASCL2(F)<br>hASCL2(R)	Human ASCL2	(F): CGT TCC GCC TAC TCG TC<br>(R): TGA GGC TCA TAG GTC GAG G
hLGR5(F)<br>hLGR5(R)	Human LGR5	(F): GGA ATG TTT CAG GCT CAA GAT G<br>(R): TGT TCA CAG GGT TTG AAG GG
hPPIA(F)<br>hPPIA(R)	Human PPIA	(F): TGC AGA CAA GGT CCC AAA G<br>(R): CCT GAC ACA TAA ACC CTG GAA TA
hB2M(F)<br>hB2M(R)	Human B2M	(F): TCG CGC TAC TCT CTC TTT CT<br>(R): TTT CCA TTC TCT GCT GGA TGA C
