# Supplementary material for: Let-7 Represses Carcinogenesis and a Stem Cell Phenotype in the Intestine via Regulation of Hmga2
Source: PLoS Genet. 2015 Aug 5;11(8):e1005408. doi: 10.1371/journal.pgen.1005408 (PMC4526516; doi:10.1371/journal.pgen.1005408)
Supplement: S1 Methods — (DOCX) [file pgen.1005408.s009.docx]

**SUPPLEMENTAL EXPERIMENTAL PROCEDURES**

**Colon Cancer Tumor Tissue Array Staining and Analysis:**

Tissue samples

Formalin-fixed paraffin-embedded (FFPE) tumors from a cohort of 227 consecutive patients with stage II and III colon adenocarcinoma submitted to curative-intent surgical resection from 1998 to 2005, receiving 5-fluorouracil (5-FU)-based adjuvant chemotherapy, and with complete follow-up in the Gastroenterology Department were obtained from Hospital Clinic Pathology Department files, Barcelona, Spain. Adjuvant chemotherapy consisted of six cycles of 5-FU (425 mg/m^2^) plus leucovorin (20 mg/m2) by rapid intravenous injection daily for 5 consecutive days every 4 weeks (in patients older than 70, the dose of 5-FU was reduced to 370 mg/m^2^). Exclusion criteria included any personal or family history of polyposis or Lynch syndromes, or personal history of inflammatory bowel disease, R1 (complete) or R2 (partial) resections (microscopic or macroscopic neoplastic involvement of surgical margins, respectively), and lack of available FFPE block. Patients with rectal cancer were also intentionally excluded since the therapeutic approach usually differs from the one employed in patients with colon cancer; indeed, most patients with rectal cancer are treated with neoadjuvant radio-chemotherapy and, based on current knowledge, molecular factors involved in the response to radiation therapy may be different to those involved in chemotherapy sensitivity/resistance. All cases were anonymized and the study was approved by the hospital Institutional Review Board and Ethics Committee.

Tissue microarray design

Twelve tissue microarrays (TMAs) were constructed with representative tumor areas from all CRC previously selected on H&E slides and marked on the corresponding paraffin blocks. Two representative 1.5 mm tissue cores were obtained from each tumor. Tissue cores were precisely arrayed in a recipient paraffin block using the automated tissue microarrayer workstation TMA Master (3DHISTHECH Ltd., Budapest, Hungary). Each TMA was stained with H&E to confirm the presence of tumor.

Immunohistochemical analysis

Protein expression was evaluated by immunohistochemistry on 2 µm sections from each TMA, mounted on charged slides, deparaffinized in xylene, and rehydrated through a graded alcohol series to distilled water. Adequate immunoreactive tissue sample was used as positive control. A negative control was obtained by omission of the primary antibodies. The immunostained sections were semi-quantitatively scored by an experienced pathologist according to a modification of the scoring by McCarty et al. [1]. The H-score ranged from 0 to 300 and was calculated by multiplying the intensity of staining (0 = no staining; 1 = weak; 2 = moderate; and 3 = strong) by the percentage of immunoreactive tumor cells (0 to 100). The H-score was obtained on the 2 tissue cores from each selected area and the mean value was considered the final H-score.

Statistical Analysis

Continuous variables were reported as mean ± standard deviation, and compared using Student’s t-test. Categorical variables were compared using the Chi-square test. Tumor HMGA1 and HMGA2 immunostaining was evaluated according to three parameters: intensity, which was recoded in low (0-1) and high (2-3); percentage of positive cells, which was recorded as negative (0-5 cells), positive + (6-50 cells), and positive ++ (51-100 cells); and the score, which was calculated as intensity * percentage of positive cells, was dichotomized according to the corresponding median value (i.e. 120 for HMGA1 and 80 for HMGA2). Probabilities of overall survival, colorectal cancer-related survival and tumor recurrence were calculated according to the Kaplan-Meier method, comparing curves by the Breslow and Mantel-Cox tests. These analyses were calculated for the whole series after adjusting by tumor stage (i.e. II vs. III). Statistical significance was set at a p-value <0.05. Statistical analysis was done using PASW Statistics 18 version 18.0.0 (SPSS Inc., Chicago, IL).

1. McCarty, K.S., Jr., et al., *Estrogen receptor analyses. Correlation of biochemical and immunohistochemical methods using monoclonal antireceptor antibodies.* Arch Pathol Lab Med, 1985. **109**(8): p. 716-21.
